# Integrative metagenomic and metabolomic analyses reveal the potential of gut microbiota to exacerbate acute pancreatitis

**DOI:** 10.1038/s41522-024-00499-4

**Published:** 2024-03-21

**Authors:** Jianjun Liu, Qiulong Yan, Shenghui Li, Juying Jiao, Yiming Hao, Guixin Zhang, Qingkai Zhang, Fei Luo, Yue Zhang, Qingbo Lv, Wenzhe Zhang, Aiqin Zhang, Huiyi Song, Yi Xin, Yufang Ma, Lawrence Owusu, Xiaochi Ma, Peiyuan Yin, Dong Shang

**Affiliations:** 1 Clinical Laboratory of Integrative Medicine, The First Affiliated Hospital of Dalian Medical University, Dalian, China; 2 College of Integrative Medicine, Dalian Medical University, Dalian, China; 3 Department of Microbiology, College of Basic Medical Sciences, Dalian Medical University, Dalian, China; 4 Puensum Genetech Institute, Wuhan, China; 5 Department of Gastrointestinal Surgery, Xijing Hospital, Fourth Military Medical University, Xi’an, China; 6 Pancreaticobiliary Centre, Department of General Surgery, The First Affiliated Hospital of Dalian Medical University, Dalian, China; 7 Department of Biotechnology, College of Basic Medical Sciences, Dalian Medical University, Dalian, China; 8 Second Affiliated Hospital, Dalian Medical University, Dalian, China

**Keywords:** Clinical microbiology, Metagenomics, Microbiota

## Abstract

Early dysbiosis in the gut microbiota may contribute to the severity of acute pancreatitis (AP), however, a comprehensive understanding of the gut microbiome, potential pathobionts, and host metabolome in individuals with AP remains elusive. Hence, we employed fecal whole-metagenome shotgun sequencing in 82 AP patients and 115 matched healthy controls, complemented by untargeted serum metabolome and lipidome profiling in a subset of participants. Analyses of the gut microbiome in AP patients revealed reduced diversity, disrupted microbial functions, and altered abundance of 77 species, influenced by both etiology and severity. AP-enriched species, mostly potential pathobionts, correlated positively with host liver function and serum lipid indicators. Conversely, many AP-depleted species were short-chain fatty acid producers. Gut microflora changes were accompanied by shifts in the serum metabolome and lipidome. Specifically, certain gut species, like enriched *Bilophila wadsworthia* and depleted *Bifidobacterium* spp., appeared to contribute to elevated triglyceride levels in biliary or hyperlipidemic AP patients. Through culturing and whole-genome sequencing of bacterial isolates, we identified virulence factors and clinically relevant antibiotic resistance in patient-derived strains, suggesting a predisposition to opportunistic infections. Finally, our study demonstrated that gavage of specific pathobionts could exacerbate pancreatitis in a caerulein-treated mouse model. In conclusion, our comprehensive analysis sheds light on the gut microbiome and serum metabolome in AP, elucidating the role of pathobionts in disease progression. These insights offer valuable perspectives for etiologic diagnosis, prevention, and intervention in AP and related conditions.

## Introduction

Acute pancreatitis (AP) stands as a prevalent gastrointestinal disorder characterized by pancreatic autodigestion. Emerging as a leading cause of hospital admissions within gastrointestinal diseases, AP records ~300,000 emergency department visits each year in the United States alone^1^. The course of AP is variable and challenging to predict early in its development; approximately one-fifth of patients progress to severe disease with a mortality rate of more than 30%^2^.

The early stage of AP is characterized by inflammatory cytokines-induced inflammation^3^. Increased intestinal permeability and subsequent translocation of gut-derived bacteria to pancreatic tissue may play a crucial role in aggravating inflammation^4^. Indeed, dysbiosis of the gut microbial community or mucosal damage in patients with AP could elevate intestinal permeability, leading to the translocation of bacteria from the gut to the blood or nearby tissues, such as the pancreas, thereby increasing the risk of pancreatic infection and exacerbating inflammation^5^. Studies have indicated that most pancreatic and extra-pancreatic organ infections originate in the gut, triggering inflammatory responses that are major contributors to “secondary attack” and increased late mortality of patients with severe AP (SAP)^6^. The underlying common causes of AP, such as biliary obstruction, alcohol misuse, and hypertriglyceridemia, may also induce changes in the gut microbiota^7–9^. Thus, it can be inferred that the gut microbiota is implicated in the occurrence and progression of AP with varying degrees of severity and etiologies.

Dysbiosis of the gut microbial community in patients with AP has been reported in previous studies utilizing amplicon sequencing^3,10,11^. These studies have revealed an increase in opportunistic *Enterobacteriaceae* pathogens in the feces of AP patients, alongside a reduction in members of *Lachnospiraceae* and *Ruminococcaceae*, as well as the anti-inflammatory probiotic *Bifidobacterium*^12,13^. Evidences indicate that gut microbiota dysbiosis could worsen the severity of acute pancreatitis. However, the exact mechanism remains unclear, and whether the microbiota participates in disease progression by altering metabolites is not fully understood. Thus, a comprehensive analysis of the differences in gut microbiota among a large sample size of AP patients with different etiologies and severity, especially based on whole-metagenome sequencing, would provide insights into the understanding of possible roles of gut microbiome through taxonomical and functional profiles. Moreover, emerging evidence indicates that microbial metabolites are implicated in the pathogenesis of pancreatic diseases^13,14^. It is essential to identify some key metabolites mediating microbial communities involved in AP exacerbation. The integration of multi-omics data from the gut microbiome, serum metabolome, and host physiology holds promise for unraveling mechanistic links between gut microbiota and disease^15^.

In this study, we conducted whole-metagenome shotgun sequencing and analysis of a large cohort of 82 AP patients and 115 healthy controls to discern variations in the gut microbiota associated with AP. We performed an integrative analysis encompassing the gut microbiome, serum metabolome (encompassing small molecule metabolites and lipids), and clinical phenotypes of AP patients, considering different etiologies and severities. This comprehensive approach, supplemented by bacterial culture and animal experiments, substantiated the notion that species enriched in AP can indeed exacerbate disease progression. Our findings offer valuable insights for the prevention and treatment of AP and related diseases.

## Results

### Clinical characteristics of AP patients

Between January 2018 and January 2020, a total of 325 patients diagnosed with AP and 136 healthy subjects were recruited for this study. Following meticulous screening according to the inclusion and exclusion criteria and in conjunction with a random selection of a subset of mild and moderately severe patients based on a computer-generated randomization method (Supplementary Fig. 1), the analysis focused on 197 fecal samples from 82 AP patients (comprising 38 mild AP [MAP], 19 moderately severe AP [MSAP], and 25 SAP) and 115 matched healthy controls. Among the patients, 51 (62.0%) had acute biliary pancreatitis (ABP), the most prevalent cause of AP in China, presenting elevated total bilirubin (TB) level, liver function indices, glucose, and triglyceride levels. Additionally, there were 12 cases of acute hyperlipidemic pancreatitis (AHP) with elevated triglycerides, 7 cases of acute pancreatitis caused by neoplasm (APN), and 12 classified as “others”, including 1 case of alcoholic pancreatitis and 11 idiopathic or unknown etiology. Further details regarding inclusion/exclusion criteria and the description of different etiological subtypes can be found in the “Methods” section. The demographic and clinical characteristics of the participants are summarized in Supplementary Table 1.

### Decreased alpha-diversity and altered overall microbial composition and function in AP patients

**Fig. 1:**
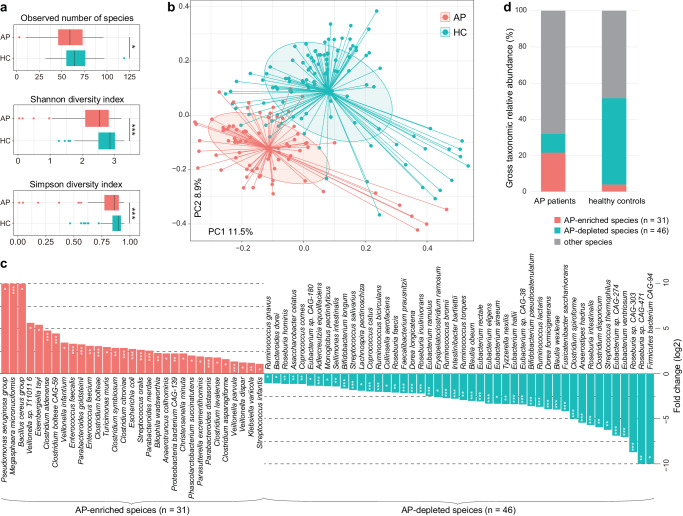
Characteristics of the gut microbial diversity and composition in AP patients. **a** Comparison of intrasample gut microbial diversity indexes between patients and controls. Boxes represent the interquartile range between the first and third quartiles and the median (internal line). Whiskers denote the lowest and highest values within 1.5 times the range of the first and third quartiles, respectively; dots represent outlier samples beyond the whiskers. Wilcoxon rank-sum test: *p < 0.05; **p < 0.01; ***p < 0.001. **b** Principal coordinates analysis (PCoA) based on the Bray–Curtis dissimilarity between microbial community composition. The result is shown in the first two principal coordinates (PC1 and PC2), and the ratios of variance contributed by these two PCs are shown. Colored points represent the samples, and circles cover samples near the center of gravity for each group. **c** Barplot showing the fold changes and *q*-values of AP-enriched and AP-depleted species. Wilcoxon rank-sum test: *q < 0.05; **q < 0.01; ***q < 0.001. **d** Gross relative abundances of AP-enriched and AP-depleted species in two groups.

To unravel the gut microbial signature of AP, we performed fecal whole-metagenome shotgun sequencing on all participants, providing profiles for 11 phyla, 25 classes, 39 orders, 78 families, 196 genera, and 618 species. Notably, AP patients exhibited a significant reduction in within-sample species richness and diversity in comparison to healthy controls (Fig. 1a). Principal coordinate analysis (PCoA) further highlighted a distinct separation between patients and controls (Fig. 1b), indicating a considerable effect size of 6.3% (permutational multivariate analysis of variance [PERMANOVA] *p* < 0.001) on the overall gut microbiota variation. Host properties such as sex, age, and body mass index showed minimal impact on the microbiota (effect size < 0.5%, PERMANOVA *p* > 0.05). Additionally, we constructed a functional profile (representing 515 metabolic pathways) of the gut microbiota. Similar to species changes, the functional diversity and composition of AP patients remarkably deviated from those in controls (effect size = 7.1%, PERMANOVA *p* < 0.001; Supplementary Fig. 2), signifying substantial dysbiosis in the holistic gut community structure and function in acute pancreatitis.

Using the false discovery rate (FDR)-corrected Wilcoxon rank-sum test, we identified 77 species that significantly differed in relative abundance between AP patients and healthy controls, with 31 species enriched in patients and 46 species depleted (*q* < 0.05 and |fold change| > 2; Fig. 1c and Supplementary Table 2). These species collectively constituted over 30% of the gross microbial abundance in both patient and control groups (Fig. 1d), underscoring their pivotal roles in the gut microbiota. Importantly, a majority (94.8%) of these species remained significant upon the MaAsLin2 algorithm^16^ with adjustment for host gender, age, and body mass index (BMI) (Supplementary Table 2). Most of these AP-enriched species, including *Escherichia coli*, *Parabacteroides* spp. (e.g., *P. distasonis*, *P. merdae*, and *P. goldsteinii*), *Veillonella* spp., and *Enterococcus* spp., have been recognized as human opportunistic pathogens in previous studies (Supplementary Table 2). Notably, *E. coli* accounted for an average relative abundance of 8.4% in patients versus 1.2% in controls. Conversely, AP-depleted primarily belonged to gut symbiotic or beneficial bacteria, involved in short-chain fatty acid (SCFAs) production in the human gut (Supplementary Table 2). Representative AP-depleted species included *Faecalibacterium prausnitzii* (average abundance in patients vs. controls, 1.6% vs. 7.0%), *Ruminococcus* (2.1% vs. 8.2%), and members of *Eubacterium* (1.7% vs. 9.9%), *Roseburia* (1.1% vs. 6.4%), and *Bifidobacterium* (0.9% vs. 4.5%).

### Etiology and severity of AP associated with the gut microbial signatures

**Fig. 2:**
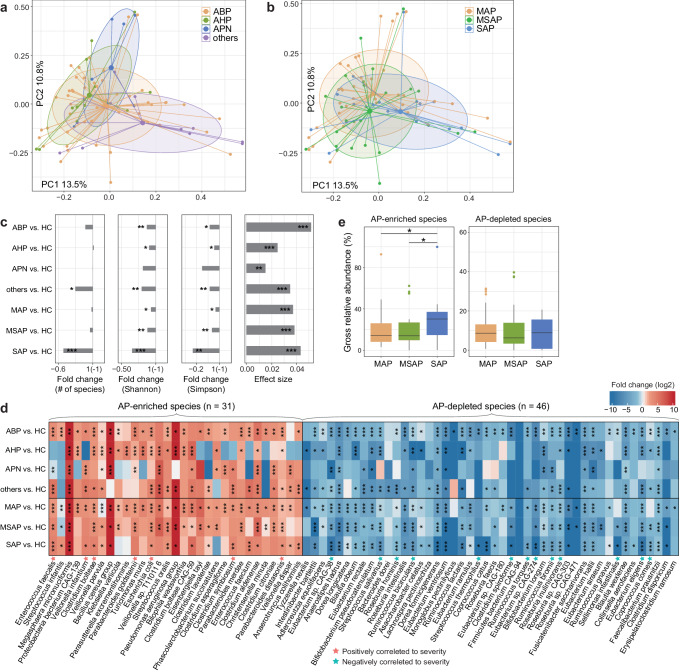
Effect of the gut microbiome by AP etiology and severity. Principal coordinates analysis (PCoA) based on the Bray–Curtis dissimilarity between microbial community composition of AP patients, samples are grouped by their etiology (**a**) and severity (**b**). The result is shown in the first two principal coordinates (PC1 and PC2), and the ratios of variance contributed by these two PCs are shown. Colored points represent the samples, and circles cover samples near the center of gravity for each group. **c** Comparison of gut microbial diversity and effect size between etiology and severity groups versus healthy controls. Left 3 panels: bar plots showing the fold changes of microbial diversity between patient groups vs. healthy controls. Statistical test is performed using Wilcoxon rank-sum test: *p < 0.05; **p < 0.01; ***p < 0.001. Right panel: bar plots showing the effect sizes on gut microbial composition for patient groups vs. healthy controls. Statistical test is performed based on PERMANOVA analysis: *, *adonis p* < 0.05; **, *adonis p* < 0.01; ***, *adonis p* < 0.001. **d** Heatmap showing the fold changes between etiology and severity groups versus healthy controls for each AP-associated species. Wilcoxon rank-sum test: *q < 0.05; **q < 0.01; ***q < 0.001. **e** Boxplot showing the total relative abundances of AP-enriched and AP-depleted species in patients with different severity. Boxes represent the interquartile range between the first and third quartiles and the median (internal line). Whiskers denote the lowest and highest values within 1.5 times the range of the first and third quartiles, respectively; dots represent outlier samples beyond the whiskers. Wilcoxon rank-sum test: *p < 0.05.

To discern the impact of AP etiology and severity on the gut microbiota, we compared the structural features of gut microbial communities in patients with different causes and severity levels. PCoA and PERMANOVA analyses revealed that significant effects of etiology (effect size = 3.1%, *p* < 0.001) and severity (effect size = 1.9%, *p* = 0.006) on the overall gut microbiota in AP patients (Fig. 2a, b), whereas other conditions, such as the time from symptom onset to admission, the time from admission to sampling, and usage of antibiotics, exhibited relatively modest influences (effect size < 0.5%, PERMANOVA *p* > 0.05 for all; Supplementary Fig. 3). Using the linear discriminant analysis (LDA) effect size (LEfSe) method^17^, we identified 21 species that differed in relative abundances among different etiologies, along with 18 species associated with severity (LDA score > 2 and *q* < 0.05; Supplementary Fig. 4 and Supplementary Table 3). Additionally, this panel of microbial species exhibited significant potential for distinguishing different causes of AP, particularly AHP (area under the receiver operating characteristic curve [AUC] = 0.752, 95% confidence interval [CI]: 0.592–0.913). It also demonstrated a notable differentiation effect concerning severity, such as SAP (AUC = 0.750, 95% CI: 0.608–0.892, Supplementary Fig. 5). These species included key members of the human gut microbiota, suggesting disease relevance of the etiology- and severity-associated signatures.

Next, we examined the relative shifts in gut microbiota for each etiology and severity group compared to healthy controls. Within-sample diversity and PERMANOVA analyses revealed substantially altered species diversity and composition in almost all groups (Fig. 2c). The degree of microbiota alterations was notably higher in patients with ABP (effect size, 5.2%) but relatively low in the AHP and APN groups (3.3% and 2.4%, respectively). Although species richness significantly decreased in SAP patients (Wilcoxon rank-sum test *p* < 0.001) compared with controls, it did not show a significant decrease in patients with MAP and MSAP. Furthermore, an analysis of 77 AP-associated species indicated reproducibility in each group versus healthy controls (Fig. 2d), suggesting a persistent major disruption of the microbiota across various facets of AP. In addition, some species showed a continuous positive (*n* = 7 AP-enriched species) or negative (*n* = 13 AP-depleted species) trend in severity from MAP to MSAP to SAP, including typical AP-enriched pathobionts (*Escherichia coli*, *Parabacteroides distasonis*, and *Enterococcus faecalis*) and AP-depleted *Streptococcus* spp. (*S. salivarius* and *S. thermophilus*) (Fig. 2d and Supplementary Fig. 6). Analyses of the gross abundances of AP-associated gut species showed a markedly higher level of AP-enriched species in SAP patients than in MAP and MSAP patients (Wilcoxon rank-sum test *q* < 0.05; Fig. 2e), suggesting a potential relationship between the load of AP pathobionts and severity.

### Correlations of the AP-associated gut species with clinical biochemical variables

**Fig. 3:**
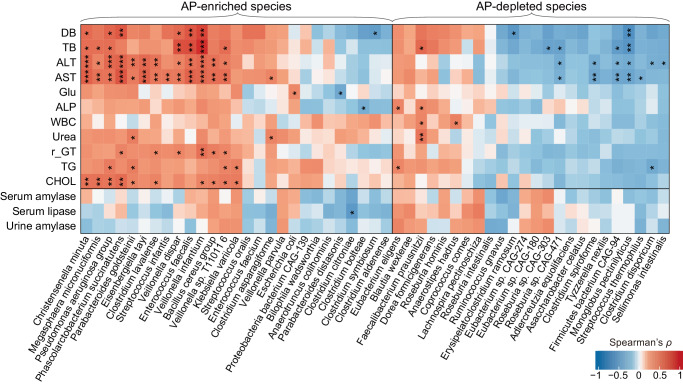
Correlations between AP-associated gut species and clinical variables. The heatmap shows the Spearman correlation coefficients between gut species and clinical variables of the AP patients. The significance level in the correlation test is denoted as: *q < 0.05; **q < 0.01; ***q < 0.001. ALT alanine transaminase, ALP alkaline phosphatase, AST aspartate aminotransferase, CHOL total cholesterol, DB direct bilirubin, γ-GT γ-glutamyl transpeptidase, Glu fasting glucose, TB total bilirubin, TG triglycerides, WBC white blood cell.

Next, we explored the inter-associations between AP-associated microbial signatures and host clinical variables using covariate-adjusted Spearman’s correlation analysis. This analysis identified numerous correlations between 31 species and all 10 investigated clinical variables (Spearman’s |*ρ*| > 0.35, *q* < 0.05; Fig. 3). Some AP-enriched species, such as *Veillonella* spp. (e.g., *V. dispar* and *V. infantium*), *Enterococcus faecalis*, and *Phascolarctobacterium succinatutens*, consistently exhibited positive correlations with liver function parameters (ALT, AST, TB, DB, γGT) and serum lipid indicators (TG, and CHOL). Conversely, several AP-depleted species, such as *Monoglobus pectinilyticus*, *Firmicutes bacterium CAG-94*, and *Roseburia sp. CAG-471*, demonstrated negative correlations with indicators of liver function. In addition, *Escherichia coli* was positively correlated with fasting blood glucose level (Spearman’s *ρ* = 0.37, *p* = 1.7 × 10^−6^; Supplementary Fig. 7), while *Faecalibacterium prausnitzii*, an anti-inflammatory bacterium that is depleted in AP patients, was positively associated with several harmful variables such as Urea, WBC, ALP, and TB; the role of these bacteria in the pathogenicity of AP requires further investigation.

Furthermore, we calculated the correlation coefficients between the AP-associated gut species and the serum digestive enzyme levels in AP patients (healthy controls were not included in this analysis) and found only one significant correlation between *Clostridium citroniae* and serum lipase (Spearman’s *ρ* = −0.36, *q* = 0.002; Fig. 3).

### Serum metabolome and lipidome reveal heightened energy metabolism expenditure in AP patients

**Fig. 4:**
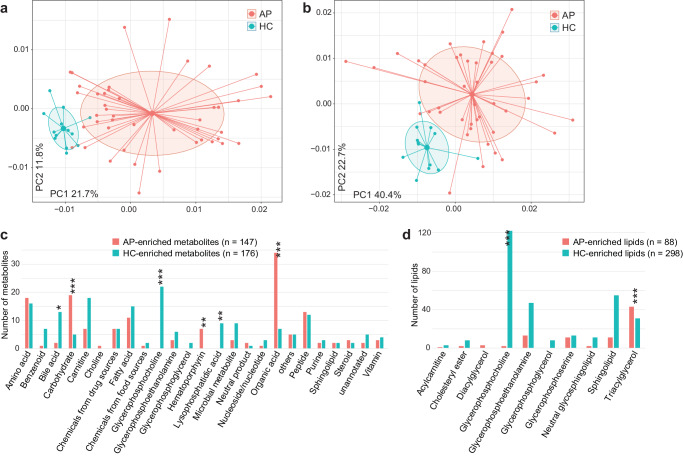
Difference in the serum metabolome and lipidome between AP patients and healthy controls. Principal coordinates analysis (PCoA) based on the Bray–Curtis dissimilarity of serum metabolome (**a**) and lipidome (**b**) between patients and controls. The result is shown in the first two principal coordinates (PC1 and PC2), and the ratios of variance contributed by these two PCs are shown. Colored points represent the samples, and circles cover samples near the center of gravity for each group. Distribution of types of AP-associated metabolites (**c**) and lipids (**d**). Statistical test is performed between AP-enriched and AP-depleted metabolites/lipids using Fisher’s exact test: *q < 0.05; **q < 0.01; ***q < 0.001.

To characterize the metabolome signatures of AP, we performed untargeted mass spectrometry (MS) profiling of serum polar metabolites and lipids in a subset of AP patients (*n* = 45, including 16 MAP, 18 MSAP, and 11 SAP patients) and healthy controls (*n* = 13). The metabolome and lipidome were characterized based on 775 annotated metabolites and 705 annotated lipids from the MS datasets, respectively. PCoA analysis illustrated significant distinctions in both the metabolome and lipidome between patients and controls (Fig. 4a, b), with disease status accounting for 17.7% (PERMANOVA, *p* < 0.001) and 22.9% (*p* < 0.001) of the variances in the metabolome and lipidome, respectively. The FDR-corrected Wilcoxon rank-sum test revealed that 41.7% of the metabolites and 54.8% of the lipids had significantly different abundances between the two groups (*q* < 0.05), while almost all of them (96.9% and 97.9%, respectively) were also significant in the MaAsLin2 test (Supplementary Tables 4 and 5). AP-enriched metabolites (*n* = 147) included a diverse array of organic acids, carbohydrates, amino acids, and hematoporphyrin, whereas HC-enriched metabolites (*n* = 176) were frequently distributed in glycerophosphocholine, carnitine, and bile acids (Fig. 4c). As for the lipidome, AP-enriched lipids (*n* = 88) were concentrated in triacylglycerols, whereas HC-enriched lipids (*n* = 298) widely spanned in glycerophosphocholines, sphingolipids, and glycerophosphoethanolamines (Fig. 4d). In patients, the altered metabolites involved in some metabolic pathways, such as arginine biosynthesis, butanoate metabolism, synthesis and degradation of ketone bodies, metabolism of aromatic amino acids, and branched-chain amino acids (Supplementary Fig. 8). These changes suggested increased energy metabolism consumption and ammonia discharge in AP, a metabolic shift documented in acute inflammation^18^. This finding corroborated the results of gut microbiota functional analysis, highlighting increased aromatic amino acid metabolism, fatty acid metabolism, ATP synthesis, and decreased carbohydrate metabolism and branched-chain amino acid metabolism (Supplementary Fig. 2b). Despite this, some AP-associated compounds, including phenylpropionylglycine, indole derivatives (e.g., indoxyl sulfate, indoleacetic acid, indole-3-propionic acid), and benzenoids (e.g., pyrocatechol sulfate), previously linked to gut bacterial fermentation of dietary substances^19,20^, exhibited changes in serum levels, potentially influenced by gut microbiota. Most of these bacteria-derived compounds were depleted in patients with AP, except for indole and phenylpropionylglycine (Fig. 4c and Supplementary Table 4).

Similar to the gut microbiome, the etiology and severity of AP considerably affected the serum metabolome and lipidome (PERMANOVA, *p* < 0.05) with minor effect sizes (Supplementary Fig. 9a, b). Among the 323 AP-associated metabolites and 386 AP-associated lipids, 59 and 15, respectively, differed in abundance among different AP etiologies, and 49 and 36, respectively, were associated with AP severity (LDA score > 2 and *q* < 0.05; Supplementary Tables 4 and 5). Among these, several molecules exhibited positive correlations with AP severity, encompassing 6 metabolites (sorbitol, mannitol, inosine, hematoporphyrin, pyrrolidinone, omeprazole sulfone) and 2 lipids. Conversely, negative correlations were observed in 14 metabolites spanning peptides, carnitine, vitamins, and organic acids, along with 13 lipids encompassing various polyunsaturated fatty acids (PUFAs), cholesteryl esters, glycerophosphocholines, and glycerophosphoethanolamines (Supplementary Fig. 9c). The roles of these molecules in the context of AP severity warrant further exploration in subsequent studies.

### AP-associated gut species correlate to serum metabolome and lipidome changes

**Fig. 5:**
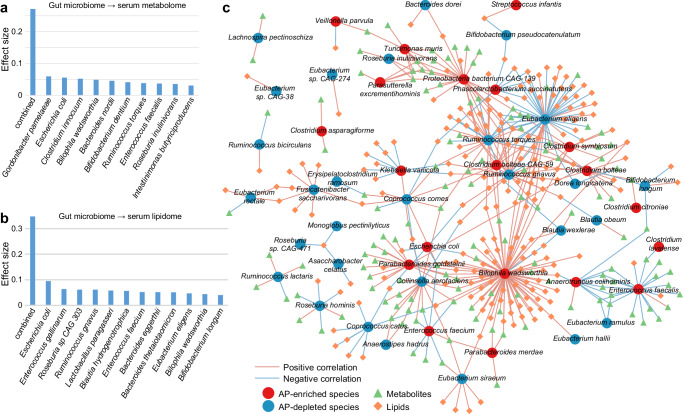
The gut microbiome influences serum metabolome and lipidome. Bar plots showing the effect size of gut species on the serum metabolome (**a**) and lipidome (**b**). Species with a significant impact (*adonis p* < 0.05) on the serum metabolome or lipidome are shown, and the combined effect sizes of all significant species for metabolome and lipidome are calculated. **c** Network showing the correlations between AP-associated gut species and serum metabolites and lipids. The Spearman correlation coefficient was used to evaluate the correlation, and correlations with an absolute correlation coefficient *ρ* > 0.35 and a correlation test *q* < 0.05 are shown in the network. Blue and red lines represent negative and positive correlations, respectively.

Next, we carried out an inter-omics PERMANOVA analysis to quantify the strength of the association between the gut microbiome, serum metabolome, and lipidome. Adonis-based univariate analysis identified 10 gut species that independently influenced the serum metabolome and 12 gut species that impacted the lipidome (Fig. 5a, b). Notably, two AP-enriched species, *Escherichia coli* and *Bilophila wadsworthia*, exhibited significant effect sizes on both the metabolome and lipidome, suggesting their important role in shaping serum metabolic homeostasis. Multivariate analysis demonstrated a substantial effect of the gut microbiome on serum metabolic profiles, explaining 27.1% and 34.1% of the variances in the metabolome and lipidome, respectively (Fig. 5a, b). This aligns with earlier findings (20–40% in patients with other diseases or special situations)^21,22^, emphasizing the significant influence of the gut microbiota on the host metabolic landscape.

To further explore the links between the gut microbiota and serum metabolome and lipidome composition, we performed an inter-omics correlation analysis involving AP-associated gut species, serum metabolites, and lipids. Using covariate-adjusted Spearman’s correlation analysis, we established a comprehensive network of 473 correlations among the species and metabolites/lipids (Spearman’s |*ρ*| > 0.35, *q* < 0.05; Fig. 5c). *Bilophila wadsworthia*, *Eubacterium eligens*, *Ruminococcus gnavus*, and *Enterococcus faecalis* emerged as key players, correlating with the highest number of metabolites and lipids (Supplementary Fig. 10). Strikingly, *Bilophila wadsworthia*, recognized as a bile-tolerant bacterium linked to animal-based diet and metabolic dysfunction^23,24^, exhibited associations with the largest number of serum metabolites and lipids, particularly various TGs (Supplementary Fig. 11a), suggesting a potential role for this bacterium in promoting the enrichment of certain detrimental substances in the blood of AP patients. In contrast, some AP-depleted bacteria, such as *Eubacterium eligens* and *Bifidobacterium* spp., displayed negative correlations with serum concentrations of TGs. Additionally, several AP-enriched species, including *Bilophila wadsworthias*, *Collinsella aerofaciens*, and *Enterococcus* spp. (*E. faecalis* and *E. faecium*), were positively associated with harmful microbial metabolites like indoxyl sulfate, p-cresol sulfate, and trimethylamine n-oxide^25–27^. This suggests that these species may contribute to the disease by affecting the concentration of metabolites in the blood (Supplementary Fig. 11b).

### The cultivated gut pathobionts from AP patients contain numerous antibiotic resistance and virulence-associated genes

To ascertain the viability of pathogens within the gut microbiota of AP patients, we cultured over 500 bacterial colonies from fresh fecal samples of a subset of randomly selected participants (19 patients and 5 controls) and sequenced their 16S rRNA genes for species identification. A total of 49 isolates corresponding to each species, including 34 AP patient-derived and 15 healthy control-derived isolates (Supplementary Table 6), were subjected to whole-genome shotgun sequencing and genomic characterization based on the uniqueness of the 16S rRNA gene within each sample. Predominant members in patient samples included *Enterococcus* spp. *Escherichia coli*, *Klebsiella* spp., and *Enterobacter* spp., whereas *Bifidobacterium* spp. and *Streptococcus salivarius* were uniquely isolated from healthy individuals (Supplementary Fig. 12a). Genomic analysis of these bacteria unveiled 481 antibiotic resistance genes (ARGs) and 121 virulence-associated genes (VAGs) (Supplementary Tables 7 and 8). Among the ARGs, 28.7% were related to intrinsic multidrug resistance (MDR), with additional genes conferring resistance against various types of clinically relevant antibiotics, including aminoglycoside (17.5%), macrolide-lincosamide-streptogramin (MLS) (11.4%), fluoroquinolone (7.1%), tetracycline (6.4%), and beta-lactamase (6.2%). Focusing on the prevalent opportunistic pathogens in AP patients, the members of Enterobacteriaceae (*E. coli*, *K. pneumoniae*, and *Citrobacter youngae*) accounted for the majority (80%) of ARGs, encompassing 81.9% MDR, 100% fluoroquinolone resistance genes, 90.0% beta-lactamase, and 91.7% sulfonamide resistance genes (Supplementary Fig. 12b). *Enterococcus faecium* and *Enterococcus faecalis* exhibited a large number of aminoglycoside and MLS resistance genes. Moreover, the majority (58.7%) of VAGs were encoded by *E. coli* and *Enterococcus faecium* strains (Supplementary Fig. 12c), with several *Enterococcus faecium* strains carrying VAGs involving fibrinogen, collagen adhesin, and cell wall-anchored proteins.

### AP patient-derived pathobionts exacerbate experimental acute pancreatitis in mice

**Fig. 6:**
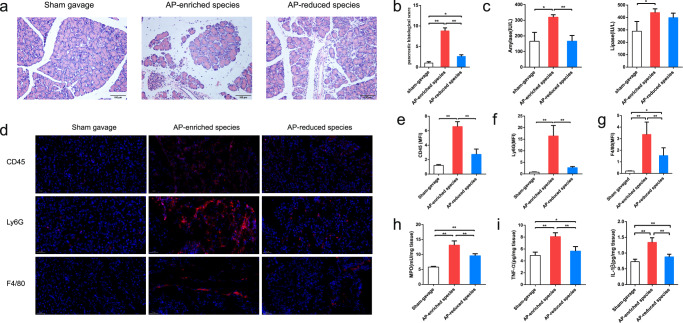
Histopathology, enzymology, and inflammatory level of the pancreas in mice after receiving AP-enriched and AP-depleted species. **a** Histological evaluation of the pancreas revealed that the pathobionts-gavaged mice had more pancreatic edema, inflammatory infiltration, and acinar cell necrosis as compared with the sham-gavaged and AP-depleted species mice groups. **b** The pancreatic histological score among the three groups. **c** The serum level of amylase (left) and lipase (right) in mice after receiving the AP-enriched and AP-depleted species. **d** Immune cells infiltrating the pancreas were characterized by immunofluorescence detection of CD45, Ly6G, and F4/80. **e**–**g** The mean fluorescence intensity (MFI) of CD45, Ly6G, and F4/80 among the three groups. **h**, **i** The myeloperoxidase (MPO) activity and the levels of proinflammatory factors TNF-α and IL-1β of the pancreas in each group. For (**b**, **c**) and (**e**–**i**), data are shown as mean ± SD. One-way ANOVA analysis: *p < 0.05; **p < 0.01.

To further test the hypothesis that the presence of AP-associated species modulates disease progression, we selected a combination of three pathobionts (*E. coli*, *Enterococcus faecalis*, and *Enterococcus faecium*) isolated from AP patients and a combination of three AP-depleted strains (*Bifidobacterium longum* and *Streptococcus salivarius* isolated from the healthy controls of this study, and *Roseburia intestinalis* isolated from a healthy subject by Zou et al.^28^) for gavage antibiotic-treated microbe-depleted mice (Supplementary Fig. 13a). After 1 week following bacterial gavage, these species were abundantly detected in the mouse intestinal tract, as shown by 16S rRNA gene sequencing of their feces and fluorescence in situ hybridization (FISH) analysis of their colonic tissues (Supplementary Fig. 13b, c). Subsequently, mice were treated with caerulein to induce the AP model. Histological evaluation of the pancreas showed pancreatic edema, massive infiltration of inflammatory cells, and a higher pancreatic tissue score in pathobiont-gavaged mice (*n* = 8) compared to AP-depleted recipient mice (*n* = 8) and sham-gavaged mice (*n* = 5). Noticeably, gavage with AP-depleted species also induced mild pancreatic inflammation above the levels observed in the sham-gavaged group (Fig. 6a, b). Furthermore, pathobiont-gavaged mice exhibited significantly higher serum amylase and lipase levels than other groups (Fig. 6c). Immunofluorescence detection of CD45, Ly6G, and F4/80 characterized immune cells infiltrating the pancreas. Pathobiont-gavaged mice showed increased infiltration of CD45-positive T cells, Ly6G-positive neutrophils, and F4/80-positive macrophages in the pancreatic tissue (Fig. 6d), and mean fluorescence intensity calculation supported these findings (Fig. 6e–g). ELISA results indicated elevated myeloperoxidase activity and higher levels of proinflammatory factors TNF-α and IL-1β in the pancreatic tissue of pathobiont-gavaged mice compared to other groups (Fig. 6h, i).

**Fig. 7:**
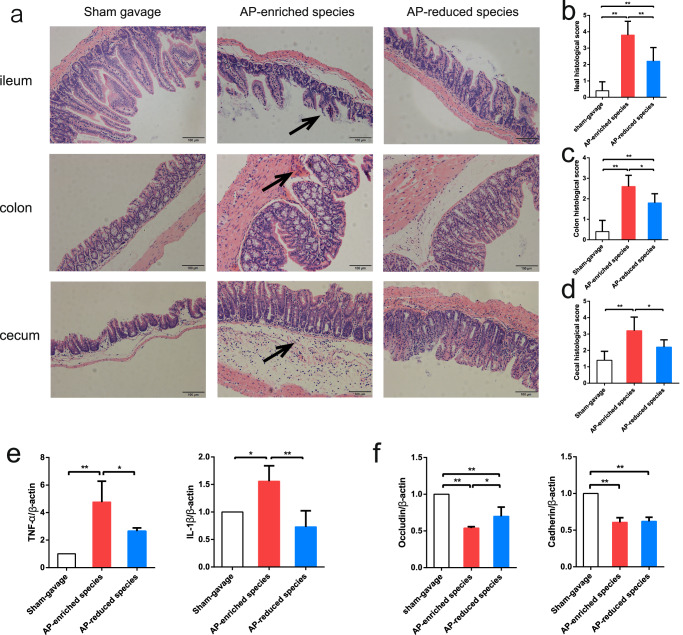
Intestinal histopathology, inflammatory factors, and intestinal barrier protein expression in mice after receiving AP-enriched and AP-depleted species. **a** Histopathology of different intestinal segments (ileum, cecum, colon) in pathobionts-gavaged mice showed that there were more inflammatory cells infiltrating the lamina propria and submucosa (as shown by the arrow), especially in the cecum, as compared with the other two groups. **b**–**d** Histopathological scores of different intestinal segments in each group. **e** The levels of proinflammatory factors TNF-α and IL-1β in the intestine among the three groups. **f** The expression of intestinal barrier proteins occludin and cadherin in the intestinal tissue. For (**b**–**f**), data are shown as mean ± SD. One-way ANOVA analysis: *p < 0.05; **p < 0.01.

Intestinal histopathology revealed more severe injury and inflammation of different intestinal segments in the pathobiont-gavaged mice than in the other groups (Fig. 7a–d). Pathobiont-gavaged mice exhibited damaged intestinal villi in the ileum, with inflammatory cell infiltration in the colon and cecum (Fig. 7a). Increased levels of pro-inflammatory factors TNF-α and IL-1β, along with reduced expression of intestinal barrier proteins occludin and cadherin, indicating compromised intestinal barrier function in the pathobiont-gavaged group (Fig. 7e, f). In summary, these findings suggest that bacteria play a crucial role in inducing acute pancreatitis, and AP-enriched pathobionts are associated with exacerbated pancreatitis progression in mice.

Additionally, we conducted a targeted metabolomic profiling on fecal samples from pathobiont-gavaged and sham-gavaged AP mice, revealing substantial differences in the fecal metabolomes between these two groups (Supplementary Fig. 14a). Pathway enrichment analysis indicated that pathobiont-gavaged mice exhibited changes in metabolic pathways resembling those observed in AP patients, including alterations in the metabolism of arginine, ketone bodies, butanoate, phenylalanine, and tyrosine (Supplementary Fig. 14b). Furthermore, when comparing the changes in fecal metabolome of pathobiont-gavaged mice with the serum metabolic alterations observed in AP patients, we identified 9 metabolites that displayed consistent trends (Supplementary Fig. 14c). Among these, 7 metabolites were upregulated in both AP patients and pathobiont-gavaged mice, including adrenal acid (a pro-inflammatory PUFA^29^), myristic acid (involving fatty acid beta-oxidation), and 3 intermediate products of the tricarboxylic acid cycle (i.e., alpha-hydrobutyric acid, oxoglutaric acid, and citromalic acid), indicating an increase in energy consumption during the disease. Conversely, 2 metabolites, gamma-linolenic acid (a PUFA with anti-inflammatory effects^30^) and ornithine, were downregulated in both AP patients and pathobiont-gavaged mice. These consistent metabolic changes suggest these AP-associated pathobionts play a synergistic role in the deterioration of disease, possibly related to energy consumption and inflammation regulation.

## Discussion

**Fig. 8:**
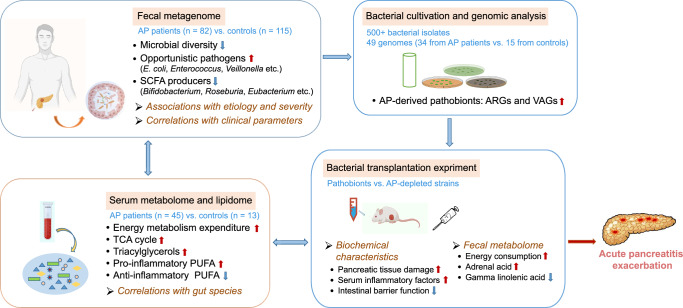
Proposed mechanism of AP exacerbation by the altered gut microbiome and serum metabolome. Flowchart depicting the integrated analyses of fecal metagenome, serum metabolome and lipidome, and bacterial cultivation and transplantation experiments employed in this study. The proposed mechanism of AP exacerbation by the altered gut microbiome and serum metabolome, as well as in bacterial transplantation mice, is shown. SCFA short-chain fatty acids, TCA cycle tricarboxylic acid cycle, PUFA polyunsaturated fatty acid, ARG antibiotic resistance gene, VAG virulence-associated gene.

In this study, we performed integrated multi-omics analyses encompassing the gut microbiome, serum metabolome, lipidome, and host phenotypes in patients with AP. Despite certain resemblances in the microflora and serum metabolites among patients with different etiologies and severities, our study adeptly identified distinguishing features related to AP etiology and disease progression. The extensive correlation observed between microbial and metabolomic profiles, coupled with the ability of cultivated gut pathobionts from AP patients to exacerbate pancreatitis in mice, provides novel insights into disease etiology (Fig. 8).

The gut microbiome of patients with AP exhibited decreased species and functional diversity compared to healthy subjects. As one of the major manifestations of gut dysbiosis, low microbial diversity frequently occurs in many diseases^31–33^ and may lead to a high risk of metabolic disorders in such individuals. Besides, the reduction in functional richness implies significant gut microbial dysfunction in AP patients.

We identified a spectrum of species with increased abundance in AP patients compared to healthy controls, including prominent species like *Escherichia coli*, *Enterococcus, Parabacteroides*, *Clostridium*, and *Veillonella* members. Notably, *E. coli*’s dominance suggests its potential role as a high-risk pathogen in AP. Literature indicates its association with severe inflammatory diseases involving the gastrointestinal tract, including Crohn’s disease^34,35^, appendicitis^36^, and peritonitis^37^. Translocation of *E. coli* from the gut to other body sites (e.g., bloodstream) is the main risk factor for infectious diseases^38^. Notably, our previous findings underscored the translocation of *E. coli* from the gut to the gallbladder, inducing acute cholecystitis^39^, further supporting its candidacy as a significant pathogen in AP and related disorders. *Enterococcus spp*., especially *E. faecalis*, exhibited a higher survival fitness in pancreatic juice^40^ and may be involved in the progression of chronic pancreatitis, as well as the initiation and advancement of pancreatic cancer^41^. Studies have identified certain bacteria elevated in the gut microbiota of AP patients, such as *Enterococcus*, *Enterobacter*, and *Clostridium* spp, which also show enrichment in that of CP patients^42,43^. CP, in turn, poses a substantial risk for the development of pancreatic cancer (PC), which established close associations with the composition of oral, fecal, and organ-specific microbiota^44^. Moreover, *E. faecalis* and *E. faecium* have been implicated as major gram-positive bacteria causing peripancreatic infections in necrotizing pancreatitis^45^. *Veillonella*, a proinflammatory gut commensal that correlates with Crohn’s disease^35^ and primary sclerosing cholangitis^46^, displayed increased abundant in AP patients. Similarly, AP-enriched *Clostridium* members, such as *C. aldenense*, *C. asparagiforme*, and *C. symbiosum*, have reported involvements in various diseases, as shown in Supplementary Table 2. Furthermore, some AP-enriched species, such as *Veillonella* spp. (e.g., *V. dispar* and *V. infantium*) and *Enterococcus faecalis*, consistently exhibited positive correlations with liver function parameters and serum lipid indicators. Other studies have also confirmed that *Veillonella* is positively correlated with ALT, AST, and TB in patients with primary sclerosing cholangitis^46^. Similarly, Duan et al. identified *Enterococcus faecalis* secreting exotoxin as a cause of hepatocyte death and liver injury^47^. Together, AP patients show significant dysbiosis of gut microbiota, likely representing the destruction of the gut-liver axis homeostasis and worsening liver function. In addition, *Escherichia coli* was positively correlated with fasting blood glucose level, in agreement with previous studies showing that gut *E. coli* abundance is a risk factor for type 2 diabetes^48,49^. Altogether, our study delineates specific species contributing to the dysbiotic gut microbiota observed in AP patients, laying the foundation for future mechanistic investigations.

Given the prevalence of opportunistic pathogens in the gut of patients with AP, we conducted bacterial cultivation research to further validated their pathogenicity. Numerous ARGs and VAGs, including extended-spectrum β-lactamases (*blaTEM-1*, *blaSHV-2*, and *blaOXA-1*)^50^ and plasmid-mediated quinolone resistance genes, were identified in patient-derived strains (e.g., *E. coli*, *Enterococcus*, *Klebsiella*, and *Parabacteroides* spp.), suggesting their potential contribution to disease pathogenesis in AP. A recent study has reported the involvement of gut microflora in the process of AP through fecal microbiota transformation in mice^12^. Here, we used gut bacterial isolates from AP patients to colonize antibiotic-treated mice. Histopathology analysis of the pancreas and serum enzymological results showed that AP-enriched pathobionts significantly exacerbated AP, while AP-depleted species recipient mice exhibited mild pancreatic injury. Inflammation in sham-gavaged mice was the mildest, indicating that the gut microbiota might serve as a stimulating factor for AP severity.

In terms of etiology and severity impact, we observed that almost all AP-enriched species accumulated in ABP, except for *V. dispar* and *Christensenella minuta*, while *Streptococcus infantis* was uniquely enriched in ABP patients but not in other etiologies. Furthermore, *Bilophila wadsworthia* was enriched in both AHP and ABP but not in others. Metabolomic analysis indicated a strong positive correlation between this bacterium and various TG. Previous studies have shown that *B. wadsworthia* is a major member of sulfidogenic bacteria in the human gut, and dietary lipids favor the growth of this pathobiont^51^. *B. wadsworthia* has been impacted in exacerbating high-fat diet-induced intestinal barrier dysfunction and bile acid dysmetabolism, leading to systemic inflammation in mice^24^. Hence, we hypothesized that *B. wadsworthia* may play a role in the pathogenesis of AHP and ABP. In addition, some AP-depleted species, such as *Eubacterium eligens*, presented a negative correlation with severity and were negatively correlated with a variety of TGs in serum, indicating that these bacteria may be involved in AP progression by regulating TG metabolism.

### Limitations of this study

Despite the robustness of our findings, there are limitations to be addressed in this study. In the process of selecting patient samples, due to the much higher incidence rates of MAP and MSAP compared to SAP, we randomly selected a portion of MAP and MSAP patients and combined all SAP individuals for subsequent multi-omics analyses. This approach may introduce a certain bias to our results regarding the microbial composition in AP patients. Future studies aiming for a more comprehensive investigation of the “natural queue” involving all patients are deemed necessary. Although the feces were collected from patients within 48 h of admission, some patients receiving intravenous antibiotic therapy (e.g., cefoperazone sulbactam) for infection on admission may experience an impact on the composition and diversity of the gut microbial community. However, the effect size was found to be < 0.5% (PERMANOVA *p* > 0.05), indicating that this short-term antibiotic exposure is unlikely to undermine our main conclusions. Additionally, the criterion of collecting fecal samples within 48 h may introduce a certain selection bias, particularly for AP patients with an ileus. Furthermore, although our patients were stratified into different severity groups, longitudinal sampling could provide valuable insights into the associations between the gut microbiome and the clinical course of the disease, particularly the onset of infected necrosis, which is a critical complication of AP likely to result from gut dysbiosis.

## Methods

### Subjects and sample collection

This study received approval from the Ethics Committee of the First Affiliated Hospital of Dalian Medical University (YJ-KS-KY-2019-93), and all participants provided written informed consent to participate in the study.

Patients aged 18–85 were recruited at an early stage (within 3 days after the onset of symptoms) of AP from the First Affiliated Hospital of Dalian Medical University and the Xijing Hospital of Airforce Medical University, China. All patients met the criteria for AP according to the revised Atlanta classification and were stratified into three groups according to clinical severity: mild (MAP, has no organ failure, local or systemic complications), moderately severe (MSAP, presence of transient organ failure, local complications, or exacerbation of co-morbid disease), and severe (SAP, organ failure lasting >48 h)^52^. Patients were excluded if they had medical histories of gastrointestinal disorders, immune deficiency, and cancers. To minimize the impact of antibiotic or other drug treatment on the gut microbiota, patients who did not provide fecal samples within 48 h after admission were excluded from the analysis. A computer-generated randomization sampling method, facilitated by the *Rv.Uniform* function in SPSS platform, was used to randomly select a subset of MAP and MSAP patients, as well as all SAP patients for further experiments (Supplementary Fig. 1). Furthermore, patients were divided into acute biliary pancreatitis (ABP), acute hyperlipidemic pancreatitis (AHP), acute alcoholic pancreatitis, acute pancreatitis caused by neoplasm (APN), and others (including idiopathic or unknown etiology) according to the etiology of the disease. The specific classification criteria were adapted from Zheng et al.’s article with slight modification^7^. Briefly, ABP was defined as the diagnosis of gallstones, cholecystitis, or alanine aminotransferase level greater than 100 U/l or total bilirubin level greater than 2.3 mg/dl. AHP was considered when the serum triglyceride level was more than 11.3 μmol/l. APN referred to AP caused by benign and malignant tumors of the pancreas. Alcoholic AP was considered in patients with confirmed alcohol consumption and a history of alcoholism, with a daily alcohol intake of more than 80 ml within 24 h before disease onset. Other AP included patients with less known causes or without any identifiable cause, such as trauma, drug use, endoscopic retrograde cholangiopancreatography, operation, sphincter of Oddi dysfunction, hypercalcemia, and autoimmune causes. Healthy controls were recruited during routine physical examinations at the Health Examination Center of the hospitals. The subjects were excluded if they had a previous medical history of gastrointestinal disorders, severe chronic metabolic diseases, cardio-cerebrovascular diseases, and cancers. Subjects who had received antibiotics or probiotics within 4 weeks prior to admission were also excluded in both patients and healthy volunteers.

All participants were provided instructions for fecal sample collection using sterile sampling tubes. Freshly collected stool samples were promptly placed in a foam box containing ice bags and transported to the laboratory within 30 min. Upon arrival, fecal samples were meticulously divided into 3–5 tubes, rapidly frozen, and stored at −80 °C until subsequent metagenomic sequencing analyses. For a subset of participants designated for bacterial cultivation, fresh fecal samples (one tube per participant) were temporarily stored at 4 °C for 1–4 h before being transferred to the culture chamber. Blood samples were collected within 24 h after admission under an 8-h fasting condition, and serum was separated by centrifugation.

### Fecal DNA extraction and whole-metagenome shotgun sequencing

Genomic DNA was extracted from fecal samples using the HiPure Stool DNA Kit (Magen, No. D3141), following the manufacturer’s guidelines. Briefly, 1 ml STL buffer was added to 50 mg of the sample in a 2-ml screw-cap tube (Axygen). Add 50 mg of zirconium oxide beads (diameter 0.5 mm). Homogenize the fecal sample in a bead-beating machine for 5 min to break up the bacteria in the feces (at 6 M/S, with 30 s of operation followed by 30 s of rest). The mixture was incubated at 65 °C for 10 min. After vortexing for 15 s and centrifuging at 13,000 × *g* for 10 min, 600 μl of the supernatant was transferred to a new 2.0-ml tube. PS buffer (150 µl) and 150 µl of absorber solution were added. Following a second centrifugation (13,000 × *g*, 5 min), the supernatants were transferred to fresh 2.0-ml tubes, and 700 µl of GDP buffer was added. A HiPure DNA Mini Column was used to absorb the products, which were eluted with sterile water. Fecal samples from all participants were subjected to whole-metagenome shotgun sequencing based on the HiSeq X platform (Illumina, USA). We constructed a 150 bp paired-end library with an insert size of ~350 bp for each sample. The initial base calling of the sequencing data was executed using the default system parameters of the sequencing platform.

### Gut metagenome analyses

The raw sequencing reads for each sample were processed for quality control using fastp^53^. Human genomic DNA reads were removed via mapping to the human reference sequences (GRCh38) with Bowtie 2^54^. The gut microbiomes were compositionally and functionally quantified using the MetaPhlAn3 and HUMAnN3 algorithms^55^, respectively. The Shannon and Simpson diversity indices, calculated using the R *vegan* package^56^, were used to represent the within-sample diversity (alpha diversity) of the microbiota in the samples. Additionally, profiles of antibiotic resistance genes were generated for each metagenomic sample by blasting the translated read sequences against the Comprehensive Antibiotic Resistance Database (CARD)^57^ using stringent cutoffs (>95% identity and >95% overlap with the query sequence).

### Serum metabolome and lipidome analyses

The serum samples were obtained from blood specimens after being centrifuged at 1500 × *g* for 10 min (4 °C), sub-packed, and stored at −80 °C for later analysis. The sample pretreatment process is as follows: first, Methanol (Fisher Scientific, Fair Lawn, USA) was added to 100 μl of the serum and vortexed for 180 s. In total, 900 μl of methyl tert-butyl ether (Sigma-Aldrich, St. Louis, USA) and 250 μl of Milli-Q (Merck KGaA, Darmstadt, Germany) purified water was then added to the solution and vortexed for 180 s. The mixture was then incubated on a rolling mixer for 10 min and kept at room temperature for 10 min, followed by centrifugation at 13,000 × *g* for 10 min (4 °C). In total, 700 μl of the lipid extract was transferred from the upper layer and 400 μl of the polar metabolite extract from the lower layer was transferred to two EP tubes, which were concentrated and dried by vacuum centrifugation. The remaining samples were mixed and centrifuged, and similarly distributed upper and lower layers were used as quality control samples. Three different analytical methods were used for polar metabolite analysis, and polar metabolite extracts were separated by reverse-phase chromatography to detect positive and negative ionization, respectively. Chromatographic separation of lipids was also carried out in positive and negative ionization modes.

Untargeted metabolome and lipidome quantifications were performed on an Ultimate 3000 ultra-high-performance liquid chromatograph and a Q Exactive quadrupole-Orbitrap high-resolution mass spectrometer (Thermo Scientific, USA). The details of chromatographic separation and mass spectrometry detection conditions for polar metabolites and lipids were described in our previous article^58^. Polar metabolites were structurally annotated by searching acquired MS2 against a local proprietary MS/MS spectrum library created using known standards, NIST 17 Tandem MS/MS library (National Institute of Standards and Technology), local version MoNA (MassBank of North America), and mzCloud library (Thermo Scientific, USA). Untargeted lipidomics data were processed using the LipidSearch software, including peak picking and lipid identification. The mass accuracies for precursor and MS/MS production searches were ±5 ppm and 5 mDa, respectively. The MS/MS similarity score threshold was set at 5.

### Bacterial cultivation and whole-genome sequencing and analyses

To isolate as many bacteria as possible from the subjects’ feces, we used 11 types of media (BHI agar, BBE agar, Bacteroides agar with 1% bile salt, YCFA, PYG, LBB, CBA agar, and BAB agar with 5% sheep blood) and aerobic/anaerobic conditions. Fresh fecal samples (200 mg) were serially diluted using physiological saline, and 200 μl suspension was plated onto Petri dishes with different solid media. These samples were then cultivated in aerobic and anaerobic incubators at 37 °C for 2–5 days. Each colony was selected and purified three times under the same conditions using a new solid medium plate. Subsequently, the liquid medium was used to conduct the purified bacterial proliferation at 37 °C for 1 day. Bacterial isolates were clarified by polymerase chain reaction via the full-length 16S rRNA gene analysis (forward primer 7F:5′-AGAGTTTGATYMTGGCTCAG-3′; reverse primer 1510R:5′-ACGGYTACCTTGTTACGACTT-3′)^59^. The centrifuged bacterial pellet was collected, and the bacterial DNA was extracted from the liquid samples, and then used for whole-genome shotgun sequencing under the HiSeq X platform (150 bp paired-end).

Short reads for each bacterial isolate were de novo assembled using the SPAdes assembler^60^ with parameters “--isolate -k 21,33,55,77”. The quality of assembled genomes was evaluated using Quast^61^. The taxonomic assignment of the genomes was generated using SpecI^62^ and whole-genome alignment against the available bacterial genomes from the National Center for Biotechnology Information (NCBI) RefSeq database (downloaded in April 2021, comprising 15,665 genomes). Gene identification was performed for all assembled genomes using Prodigal^63^. The phylogenetic tree was generated using PhyloPhlAn2^64^ and visualized using interactive Tree Of Life (iTOL)^65^. Antibiotic resistance genes and virulence-associated genes were identified using ABRicate (https://github.com/tseemann/abricate)^66^. ABRicate searched the NCBI Bacterial Antimicrobial Resistance Reference Gene Database, CARD^57^, ARG-ANNOT^67^, and ResFinder^68^ for predicting antibiotic resistance genes (ARGs), and VFDB^69^ for predicting virulence-associated genes (VAGs).

### Animal experiment

All animal experiments were performed in accordance with the recommendations of the Guide for the Care and Use of Laboratory Animals of the National Institute of Health. The animal protocols were approved by the Committee on the Ethics of Animal Experiments of Dalian Medical University (No. AEE19001). Six-week-old male C57BL/6 mice obtained from the Specific Pathogen Free Animal Center of Dalian Medical University were randomly divided into three groups: AP-enriched pathobionts (*n* = 8), AP-depleted species (*n* = 8), and sham-gavaged (*n* = 5). Before bacterial transplantation, we treated mice with broad-spectrum antibiotics (ampicillin 1 g/l, neomycin 1 g/l, metronidazole 1 g/l, and vancomycin 0.5 g/l) in their drinking water for 4 weeks, a regimen previously used to achieve bowel sterilization^70^. After antibiotic treatment, mice from each experimental group were housed in separate cages. AP-enriched pathobionts (*E. coli*, *Enterococcus faecalis*, and *Enterococcus faecium*) and AP-depleted species (*Bifidobacterium longum*, *Streptococcus salivarius*, and *Roseburia intestinalis*) were administered intragastrically once a day for 1 week, suspended in 200 µl saline with 10^7^ colony-forming units (CFU) of each species. Sham-gavaged mice were administered saline only and then challenged with repeated hourly intraperitoneal (i.p.) injections of high doses of caerulein (50 mg/kg, Sigma) seven times to induce AP models^71,72^. At 24 h after the first injection of caerulein, the mice were anesthetized with inhaled isoflurane using a gas anesthesia machine, sera, pancreatic tissue, and different intestinal segments (ileum, cecum, and colon) were obtained and sacrificed animals after sampling.

#### Serum detection

Serum was obtained by centrifuging whole blood samples. Serum level of Amylase (AMY) and Lipase (LPS) was measured using Mouse Amylase and Lipase enzyme-linked immunosorbent assay (ELISA) kit (Mlbio, Shanghai, China) according to manufacturer’s instructions. The levels of AMY and LPS in serum were determined using Microplate Reader (BioTek, USA).

#### Histological analysis

Formaldehyde-fixed pancreas and different intestinal segments were embedded in paraffin, sectioned into slices, and stained with hematoxylin-eosin (HE). Pancreatic histological score was evaluated according to Rongione’s standard^73^; Ileal mucosal injury was assessed according to Chiu’s score^74^; Geboes score (GS) was employed for histological assessment of inflammation in cecum and colon^75^. All tissue sections were determined under a light microscope (Olympus, Tokyo, Japan) by a pathologist who was blinded to the sample identity to evaluate the damage and inflammation from five random fields of each sample.

#### ELISA assay

Pancreas were homogenized in liquid nitrogen for detection of myeloperoxidase (MPO) activity and the levels of proinflammatory factors TNF-α and IL-1β with commercial ELISA kits (Mouse MPO, TNF-α and IL-1β ELISA Kit, Jiangsu Meibiao Biotechnology Co., Ltd, China) according to the manufacturer’s guidelines.

#### Immunofluorescence

In order to evaluate the infiltration of inflammatory cells in pancreas, we detected CD45, Ly6G and F4/80 by immunofluorescence. Briefly, Frozen slides are baked in a 37 °C oven for 10–20 min and fix in paraformaldehyde for 30 min. Afterward, the slides were heated in an autoclave within EDTA antigen retrieval buffer (pH 8.0) for antigen repairing, followed by 3% BSA to block non-specific binding. Slides were then incubated with primary antibody including CD45 (GB11066, Servicebio, China, 1:1000), Ly6G (GB11229, Servicebio, China, 1:500) and F4/80 (GB113373, Servicebio, China, 1:500) at 4 °C overnight, and with Cy3 conjugated secondary antibody (GB21303, Servicebio, China, 1:500) at room temperature for 50 min in dark condition. After DAPI counterstain in nucleus, the sections were observed and images collected under the fluorescence microscope. The mean fluorescence intensity (MFI) was analyzed by ImageJ (V1.8.0.112). Five visual fields were randomly selected on each slide for MFI analysis.

#### Quantification RT-PCR

Total RNA of intestinal tissue was extracted using the TRIzol Reagent (Invitrogen, USA). RNA quality was determined by a nanodrop (Thermo, USA) and reverse transcribed using an Evo M-MLV Mix Kit with gDNA Clean for qPCR (Accurate Biology; Hunan, China), which was performed using SYBR Green Premix Pro Taq HS qPCR Kit (Accurate Biology; Hunan, China) in the ABI 7500 Real-Time PCR System (Applied Biosystems; CA, USA). Primers for PCR included the following: β-actin 5’-CTACCTCATGAAGATCCTGACC-3’ and 5’-CACAGCTTCTCTTTGATGTCAC-3’; TNF-α 5’-ATGTCTCAGCCTCTTCTCATTC-3’ and 5’-GCTTGTCACTCGAATTTTGAGA-3’^13^; IL-1β 5’-CACTACAGGCTCCGAGATGAACAAC-3’ and 5’-TGTCGTTGCTTGGTTCTCCTTGTAC-3’; occludin 5’-CTCTCAGCCAGCGTACTCTT-3’ and 5’-CTCCATAGCCACCTCCGTAG-3’^13^; cadherin 5’-CCTGTCTTCAACCCAAGCAC-3’ and 5’-CAACAACGAACTGCTGGTCA-3’. The PCR procedure involved two-stage standard amplification, including 1 cycle of predenaturation at 95 °C for 30 s and 40 cycles of denaturation, annealing and extension at 95 °C for 5 s and 60 °C for 34 s. The cycle threshold (CT) values were used to calculate the relative mRNA expression through the 2^−ΔΔCT^ method.

#### Fluorescence in situ hybridization (FISH)

Colonic tissue sections underwent processing to optimize conditions for FISH. Initially, sections were baked at 70 °C for 1 h to ensure proper adhesion. A paraffin deparaffinization step was performed by immersing sections into a preheated dewaxing agent at 68 °C for 15 min. Subsequent washing at room temperature in 100% ethanol for 5 min removed residual paraffin. Permeabilization was achieved by immersing sections into a EDTA solution at 90 °C for 20 min to enhance the penetration of probes. Further washing at 37 °C in deionized water for 3 min followed permeabilization. For enzymatic digestion, sections were submerged in a preheated pepsin working solution at 37 °C for 10 min. Post-digestion, sections were washed twice in wash solution (2 × SSC) for 5 min each. Dehydration involved sequential immersion in 70%, 85%, and 100% ethanol for 2 min each, concluding with air-drying at room temperature. The probe sequences used in the FISH experiments were as follows: FAM-conjugated EUB338 universal bacterial probe (GCTGCCTCCCGTAGGAGT)^76^, and Cy3-conjugated specific probes targeting *Streptococcus salivarius* (GATGACGAATAGGTGTTAG), *Roseburia intestinalis* (ATTAGAGGCGGTCAAGAA), *Bifidobacterium longum* (CATCATCAACACTGAATCG), *E. coli* (ACGTCAATGAGCAAAGG) and *Enterococcus sp*. (GTTCTCTGCGTCTACCTC)^77^. Probes (6 μl) were evenly applied to the hybridization region, sealed, and subjected to denaturation at 85 °C for 5 min, followed by hybridization at 42 °C for 2–4 h. Post-hybridization, cover slips were removed, and sections were sequentially immersed in 2 × SSC for 1 min, hybridization post-wash solution at 68 °C for 2 min, deionized water at 37 °C for 1 min, and air-dried in darkness. The hybridization region was counterstained with DAPI staining solution, covered with a glass coverslip, and left undisturbed in darkness for 10 min. Prepared slides were observed using a fluorescence microscope.

### Statistical analyses

Statistical analyses were performed using the R v3.3.2. Permutational multivariate analysis of variance (PERMANOVA) was performed with the *adonis* function of the R *vegan* package, and the *adonis p* value was generated based on 1000 permutations. The effect size (R2) was calculated from the *adonis* analysis. The *adonis*-based univariate and multivariate analyses on the microbiota dataset were performed following the methods developed by Wang et al.’s study^21^. Principal coordinate analysis (PCoA) and distance-based redundancy analysis (dbRDA) were performed based on the Bray–Curtis dissimilarity of the gut microbial composition and functional profile using *capscale* function (*vegan* package). For comparison analyses, the *p* values were calculated using the Wilcoxon rank-sum test, the linear discriminant analysis (LDA) effect size (LEfSe) method^17^, and the MaAsLin2 algorithm^16^ with adjustment for host gender, age, and BMI. *q* was used to evaluate the false discovery rate (FDR) for correction of multiple comparisons and was calculated using the R *fdrtool* package. Statistical scripts are available at https://github.com/lish2/ap_microbiome.

### Supplementary information

Supplementary Fig. 1-14

Supplementary Table 1-8

reporting-summary

## Data Availability

The raw whole-metagenomic shotgun sequencing dataset acquired in this study has been deposited in the European Bioinformatics Institute (EBI) database under the accession code PRJEB36300. The assembled bacterial genome sequences reported in this article were deposited in the NCBI BioProject PRJNA612981. The metabolome datasets reported in this article were available at the MetaboLights database (https://www.ebi.ac.uk/metabolights/) with accession number MTBLS9696. Other data related to the current article are available from the corresponding author on reasonable request.

## References

[1] Mederos MA, Reber HA, Girgis MD (2021). Acute pancreatitis: a review. JAMA.

[2] Forsmark CE, Vege SS, Wilcox CM (2016). Acute pancreatitis. N. Engl. J. Med..

[3] Li XY, He C, Zhu Y, Lu NH (2020). Role of gut microbiota on intestinal barrier function in acute pancreatitis. World J. Gastroenterol..

[4] Wu L (2023). Gut microbiota interacts with inflammatory responses in acute pancreatitis. Ther. Adv. Gastroenterol..

[5] Ahuja M (2017). Orai1-mediated antimicrobial secretion from pancreatic acini shapes the gut microbiome and regulates gut innate immunity. Cell Metab..

[6] Ammori BJ (2003). Role of the gut in the course of severe acute pancreatitis. Pancreas.

[7] Zheng Y (2015). A multicenter study on etiology of acute pancreatitis in Beijing during 5 years. Pancreas.

[8] Zhu Y (2017). A study on the etiology, severity, and mortality of 3260 patients with acute pancreatitis according to the revised Atlanta classification in Jiangxi, China over an 8-year period. Pancreas.

[9] Zilio MB, Eyff TF, Azeredo-Da-Silva ALF, Bersch VP, Osvaldt AB (2019). A systematic review and meta-analysis of the aetiology of acute pancreatitis. HPB.

[10] Tan C (2015). Dysbiosis of intestinal microbiota associated with inflammation involved in the progression of acute pancreatitis. Pancreas.

[11] Yu S (2020). Identification of dysfunctional gut microbiota through rectal swab in patients with different severity of acute pancreatitis. Dig. Dis. Sci..

[12] Zhu Y (2019). Gut microbiota dysbiosis worsens the severity of acute pancreatitis in patients and mice. J. Gastroenterol..

[13] van den Berg, F. F. et al. Western-type diet influences mortality from necrotising pancreatitis and demonstrates a central role for butyrate. *Gut* **70**, 915–927 (2020). 10.1136/gutjnl-2019-320430 PMC7917160 32873697

[14] Tran QT (2021). Role of bile acids and bile salts in acute pancreatitis: from the experimental to clinical studies. Pancreas.

[15] Pedersen HK (2018). A computational framework to integrate high-throughput ‘-omics’ datasets for the identification of potential mechanistic links. Nat. Protoc..

[16] Mallick H (2021). Multivariable association discovery in population-scale meta-omics studies. PLoS Comput. Biol..

[17] Segata N (2011). Metagenomic biomarker discovery and explanation. Genome Biol..

[18] Varela ML, Mogildea M, Moreno I, Lopes A (2018). Acute inflammation and metabolism. Inflammation.

[19] Wikoff WR (2009). Metabolomics analysis reveals large effects of gut microflora on mammalian blood metabolites. Proc. Natl Acad. Sci. USA.

[20] Konopelski P, Mogilnicka I (2022). Biological effects of indole-3-propionic acid, a gut microbiota-derived metabolite, and its precursor tryptophan in mammals’ health and disease. Int. J. Mol. Sci..

[21] Wang X (2020). Aberrant gut microbiota alters host metabolome and impacts renal failure in humans and rodents. Gut.

[22] Sang X (2023). Dynamics and ecological reassembly of the human gut microbiome and the host metabolome in response to prolonged fasting. Front. Microbiol.

[23] David LA (2014). Diet rapidly and reproducibly alters the human gut microbiome. Nature.

[24] Natividad JM (2018). Bilophila wadsworthia aggravates high fat diet induced metabolic dysfunctions in mice. Nat. Commun..

[25] Lano, G., Burtey, S. & Sallee, M. Indoxyl sulfate, a uremic endotheliotoxin. *Toxins* **12**, 229 (2020). 10.3390/toxins12040229 PMC7232210 32260489

[26] Croci, S., D’Apolito, L. I., Gasperi, V., Catani, M. V. & Savini, I. Dietary strategies for management of metabolic syndrome: role of gut microbiota metabolites. *Nutrients* **13**10.3390/nu13051389 (2021). 10.3390/nu13051389 PMC8142993 33919016

[27] Li D (2022). Gut microbiota-derived metabolite trimethylamine-N-oxide and multiple health outcomes: an umbrella review and updated meta-analysis. Am. J. Clin. Nutr..

[28] Zou Y (2019). 1520 reference genomes from cultivated human gut bacteria enable functional microbiome analyses. Nat. Biotechnol..

[29] Zhao S (2022). Alteration of bile acids and omega-6 PUFAs are correlated with the progression and prognosis of drug-induced liver injury. Front. Immunol..

[30] Sergeant S, Rahbar E, Chilton FH (2016). Gamma-linolenic acid, dihommo-gamma linolenic, eicosanoids and inflammatory processes. Eur. J. Pharm..

[31] Kuang YS (2017). Connections between the human gut microbiome and gestational diabetes mellitus. Gigascience.

[32] Yan Q (2017). Alterations of the gut microbiome in hypertension. Front. Cell Infect. Microbiol..

[33] Lv LJ (2019). Early-onset preeclampsia is associated with gut microbial alterations in antepartum and postpartum women. Front. Cell Infect. Microbiol..

[34] Sasaki M (2007). Invasive Escherichia coli are a feature of Crohn’s disease. Lab. Invest..

[35] Gevers D (2014). The treatment-naive microbiome in new-onset Crohn’s disease. Cell Host Microbe.

[36] Juric I (2001). Frequency of portal and systemic bacteremia in acute appendicitis. Pediatr. Int..

[37] Roehrborn A (2001). The microbiology of postoperative peritonitis. Clin. Infect. Dis..

[38] Kaper JB, Nataro JP, Mobley HL (2004). Pathogenic Escherichia coli. Nat. Rev. Microbiol..

[39] Liu J (2015). Acute cholecystitis associated with infection of Enterobacteriaceae from gut microbiota. Clin. Microbiol. Infect..

[40] Itoyama S (2022). Enterococcus spp. have higher fitness for survival, in a pH‐dependent manner, in pancreatic juice among duodenal bacterial flora. JGH Open.

[41] Maekawa T (2018). Possible involvement of Enterococcus infection in the pathogenesis of chronic pancreatitis and cancer. Biochem. Biophys. Res. Commun..

[42] Frost F (2020). The Gut Microbiome in Patients With Chronic Pancreatitis Is Characterized by Significant Dysbiosis and Overgrowth by Opportunistic Pathogens. Clin Transl Gastroenterol..

[43] Hamada S, Masamune A, Nabeshima T, Shimosegawa T (2018). Differences in gut microbiota profiles between autoimmune pancreatitis and chronic pancreatitis. Tohoku J. Exp. Med..

[44] Thomas, R. M. & Jobin, C. Microbiota in pancreatic health and disease: the next frontier in microbiome research. *Nat. Rev. Gastroenterol. Hepatol*. **17**, 53–64 (2020). 10.1038/s41575-019-0242-7 31811279

[45] Tian H (2020). Infectious complications in severe acute pancreatitis: pathogens, drug resistance, and status of nosocomial infection in a university-affiliated teaching hospital. Dig. Dis. Sci..

[46] Liu Q (2022). Altered faecal microbiome and metabolome in IgG4-related sclerosing cholangitis and primary sclerosing cholangitis. Gut.

[47] Duan Y (2019). Bacteriophage targeting of gut bacterium attenuates alcoholic liver disease. Nature.

[48] Wu H (2017). Metformin alters the gut microbiome of individuals with treatment-naive type 2 diabetes, contributing to the therapeutic effects of the drug. Nat. Med..

[49] Zhong H (2019). Distinct gut metagenomics and metaproteomics signatures in prediabetics and treatment-naïve type 2 diabetics. EBioMedicine.

[50] Hansen KH (2019). Resistance to piperacillin/tazobactam in Escherichia coli resulting from extensive IS 26-associated gene amplification of bla TEM-1. J. Antimicrob. Chemother..

[51] Feng Z (2017). A human stool-derived Bilophila wadsworthia strain caused systemic inflammation in specific-pathogen-free mice. Gut Pathog..

[52] Banks PA (2013). Classification of acute pancreatitis–2012: revision of the Atlanta classification and definitions by international consensus. Gut.

[53] Chen S, Zhou Y, Chen Y, Gu J (2018). fastp: an ultra-fast all-in-one FASTQ preprocessor. Bioinformatics.

[54] Langmead B, Salzberg SL (2012). Fast gapped-read alignment with Bowtie 2. Nat. Methods.

[55] Beghini, F. et al. Integrating taxonomic, functional, and strain-level profiling of diverse microbial communities with bioBakery 3. *Elife* **10**10.7554/eLife.65088 (2021). 10.7554/eLife.65088 PMC8096432 33944776

[56] Dixon P (2003). VEGAN, a package of R functions for community ecology. J. Veg. Sci..

[57] Jia B (2017). CARD 2017: expansion and model-centric curation of the comprehensive antibiotic resistance database. Nucleic Acids Res..

[58] Deng, D. et al. An integrated metabolomic study of osteoporosis: discovery and quantification of hyocholic acids as candidate markers. *Front. Pharmacol.* **12**, 725341 (2021). 10.3389/fphar.2021.725341 PMC8378234 34421618

[59] Browne HP (2016). Culturing of ‘unculturable’ human microbiota reveals novel taxa and extensive sporulation. Nature.

[60] Bankevich A (2012). SPAdes: a new genome assembly algorithm and its applications to single-cell sequencing. J. Comput. Biol..

[61] Gurevich A, Saveliev V, Vyahhi N, Tesler G (2013). QUAST: quality assessment tool for genome assemblies. Bioinformatics.

[62] Mende DR, Sunagawa S, Zeller G, Bork P (2013). Accurate and universal delineation of prokaryotic species. Nat. Methods.

[63] Hyatt D (2010). Prodigal: prokaryotic gene recognition and translation initiation site identification. BMC Bioinforma..

[64] Segata N, Bornigen D, Morgan XC, Huttenhower C (2013). PhyloPhlAn is a new method for improved phylogenetic and taxonomic placement of microbes. Nat. Commun..

[65] Letunic I, Bork P (2019). Interactive Tree Of Life (iTOL) v4: recent updates and new developments. Nucleic Acids Res..

[66] Seemann, T. Abricate. *Github* https://github.com/tseemann/abricate (2020).

[67] Gupta SK (2014). ARG-ANNOT, a new bioinformatic tool to discover antibiotic resistance genes in bacterial genomes. Antimicrob. Agents Chemother..

[68] Zankari E (2012). Identification of acquired antimicrobial resistance genes. J. Antimicrob. Chemother..

[69] Chen L (2005). VFDB: a reference database for bacterial virulence factors. Nucleic Acids Res..

[70] Rakoff-Nahoum S, Paglino J, Eslami-Varzaneh F, Edberg S, Medzhitov R (2004). Recognition of commensal microflora by toll-like receptors is required for intestinal homeostasis. Cell.

[71] Sharif R (2009). Impact of toll-like receptor 4 on the severity of acute pancreatitis and pancreatitis-associated lung injury in mice. Gut.

[72] Tsuji Y (2012). Sensing of commensal organisms by the intracellular sensor NOD1 mediates experimental pancreatitis. Immunity.

[73] Rongione AJ (1997). Interleukin 10 reduces the severity of acute pancreatitis in rats. Gastroenterology.

[74] Chiu CJ, McArdle AH, Brown R, Scott HJ, Gurd FN (1970). Intestinal mucosal lesion in low-flow states. I. A morphological, hemodynamic, and metabolic reappraisal. Arch. Surg..

[75] Geboes K (2000). A reproducible grading scale for histological assessment of inflammation in ulcerative colitis. Gut.

[76] Long X (2019). Peptostreptococcus anaerobius promotes colorectal carcinogenesis and modulates tumour immunity. Nat. Microbiol..

[77] Azevedo AS (2022). Spectral imaging and nucleic acid mimics fluorescence in situ hybridization (SI-NAM-FISH) for multiplex detection of clinical pathogens. Front. Microbiol..

