# Supplementary material for: Integrative metagenomic and metabolomic analyses reveal the potential of gut microbiota to exacerbate acute pancreatitis
Source: NPJ Biofilms Microbiomes. 2024 Mar 21;10:29. doi: 10.1038/s41522-024-00499-4 (PMC10957925; doi:10.1038/s41522-024-00499-4)
Supplement: Supplementary file 1 — Supplementary Fig. 1-14 [file 41522_2024_499_MOESM1_ESM.pdf]

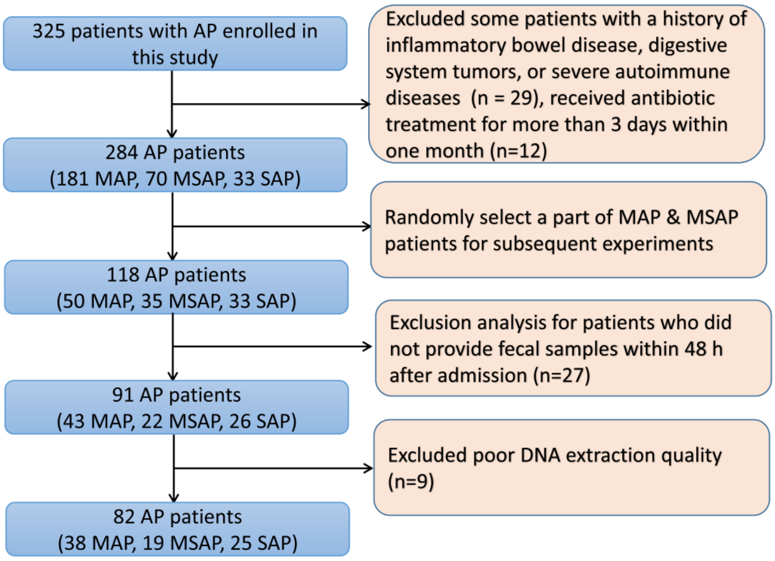

Supplementary Fig. 1| Flowchart illustrating the recruitment process of patients with acute pancreatitis in this study. AP, acute pancreatitis; MAP, mild acute pancreatitis; MSAP, moderately severe acute pancreatitis; SAP, severe acute pancreatitis. All patients met the criteria for AP according to the revised Atlanta classification and were stratified into three groups (MAP, MSAP, and SAP) according to clinical severity. Patients were excluded if they had medical histories of gastrointestinal disorders, immune deficiency, and cancers etc. A computer-generated random sampling method based on the SPSS software was used to randomly select a part of MAP and MSAP, as well as all SAP patients for further analysis. To minimize the impact of antibiotic or other drug treatment on the gut microbiota, patients who did not provide fecal samples within 48 hours after admission were excluded from the analysis.

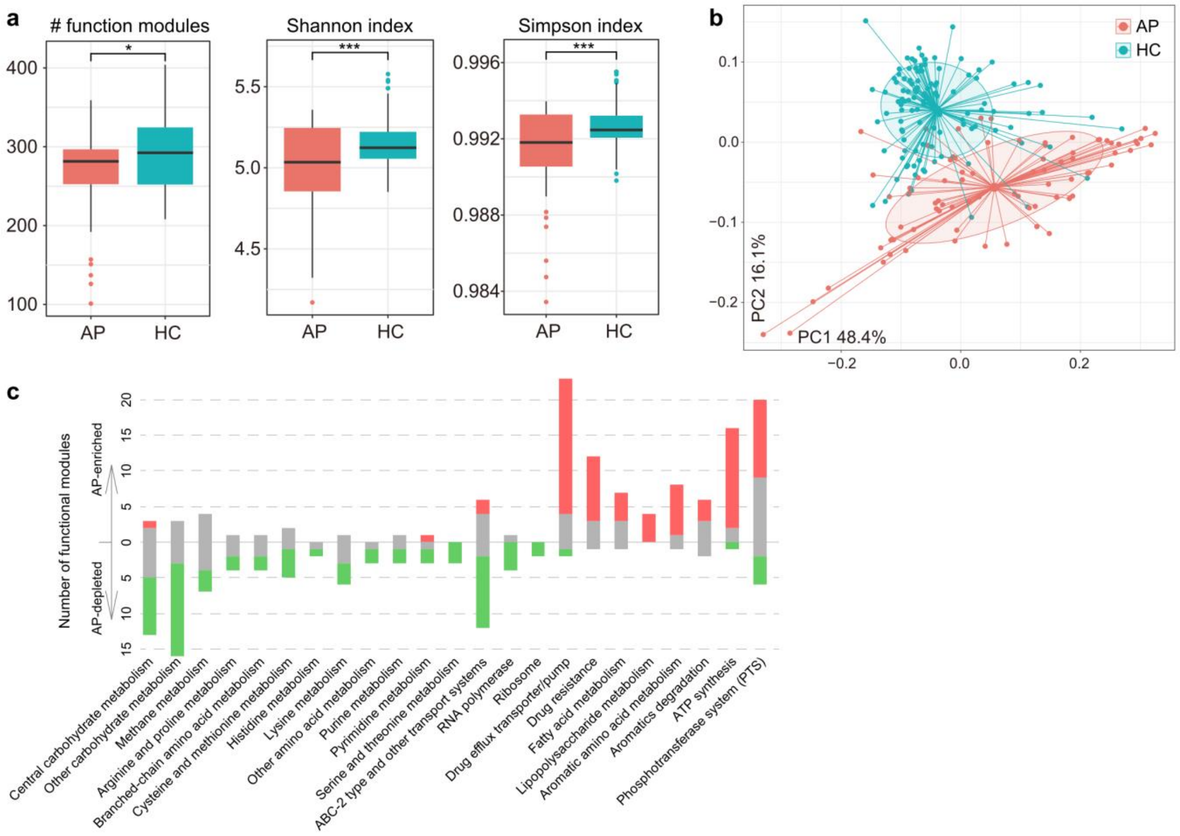

Supplementary Fig. 2| Comparison of gut microbial functions between AP patients and healthy controls. (a) Comparison of intrasample microbial functional diversity indexes between patients and controls. Boxes represent the interquartile range between the first and third quartiles and the median (internal line). Whiskers denote the lowest and highest values within 1.5 times the range of the first and third quartiles, respectively; dots represent outlier samples beyond the whiskers. Wilcoxon rank-sum test: *, *p*<0.05; **, *p*<0.01; ***, *p*<0.001. **(b)** Principal coordinates analysis (PCoA) based on the Bray-Curtis dissimilarity between microbial functional profiles. The result is shown in the first two principal coordinates (PC1 and PC2), and the ratios of variance contributed by these two PCs are shown. Colored points represent the samples, and circles cover samples near the center of gravity for each group. **(c)** Comparison of functional modules between AP patients and healthy controls. Functional modules are categorized at KEGG level B, and the barplot shows the number of modules (in each category) that enriched in patients or controls. Colors represent enrichment in patients or controls: red, AP-enriched; green, AP-depleted; gray, not significant.

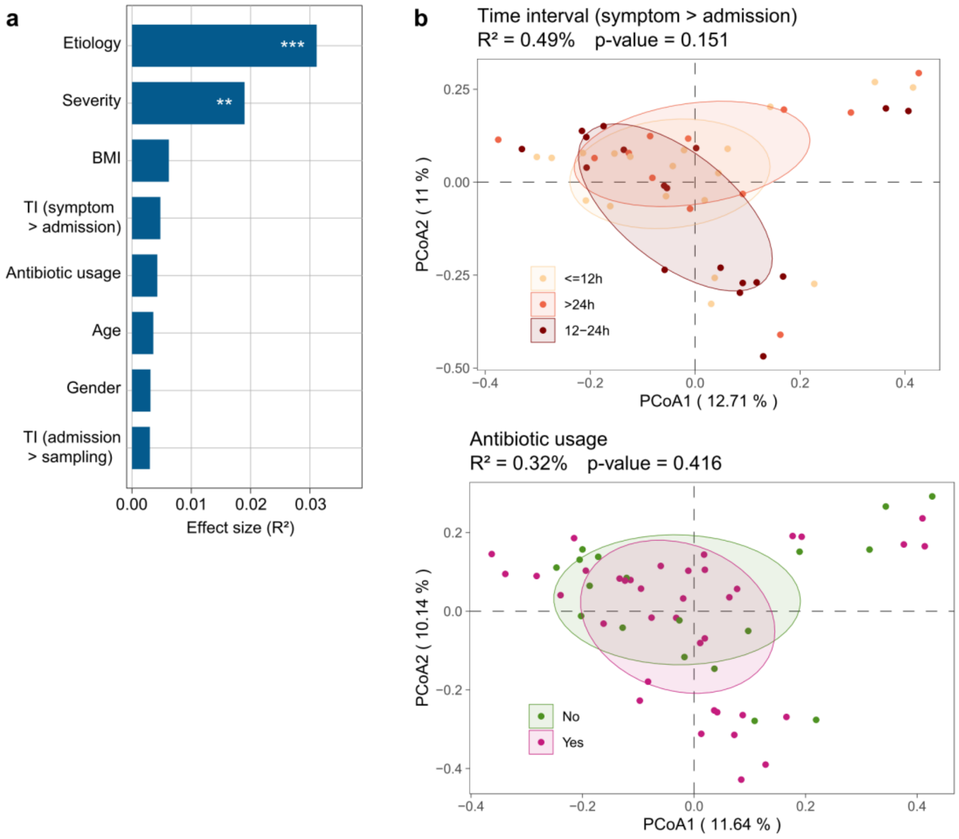

Supplementary Fig. 3| Influence of the gut microbiota by clinical conditions of patients. (a) Bar plots showing the effect sizes (R^2) on gut microbial composition by clinical conditions. Statistical test is performed based on PERMANOVA analysis: *, *adonis p*<0.01; ***, *adonis p*<0.001. **(b)** Principal coordinates analysis (PCoA) based on the Bray-Curtis dissimilarity between microbial community composition of AP patients, samples are grouped by their time from symptom to admission (upper panel) and antibiotic usage (bottom panel).

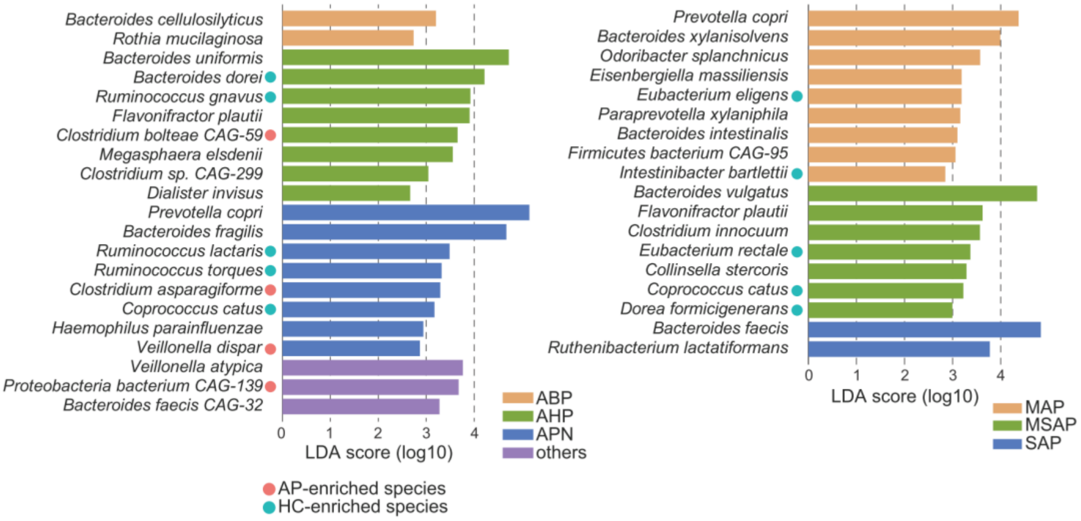

Supplementary Fig. 4| Alterations of the gut microbial composition among different AP etiology and severity. Barplots showing the differential species that significantly differed in relative abundance among different etiology (left panel) and severity (right panel). The species are identified based on the linear discriminant analysis (LDA) effect size (LEfSe) algorithm, with an LDA score >2 and $q < 0.05$. Species that are enriched or depleted in AP patients versus healthy controls are shown.

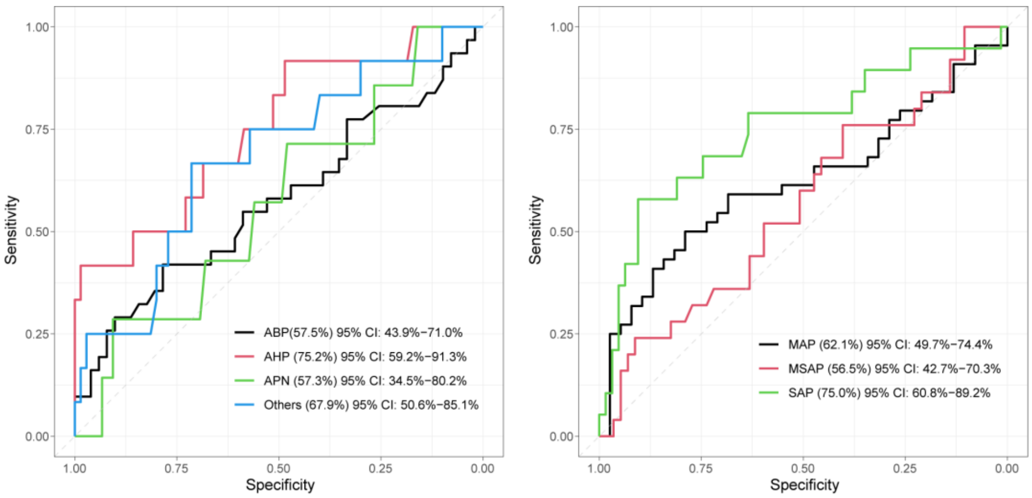

Supplementary Fig. 5| Predictive microbiome signatures of AP etiology and severity. Receiver operator characteristic (ROC) analysis for distinguishing different causes and severity of AP based on the set of 21 and 18 microbial species above respectively. Random forest models were analyzed using the R *randomForest* package (1,000 trees). The performance of the predictive model was evaluated using leave-one-out cross validation. ROC analysis was performed using the R *pROC* package.

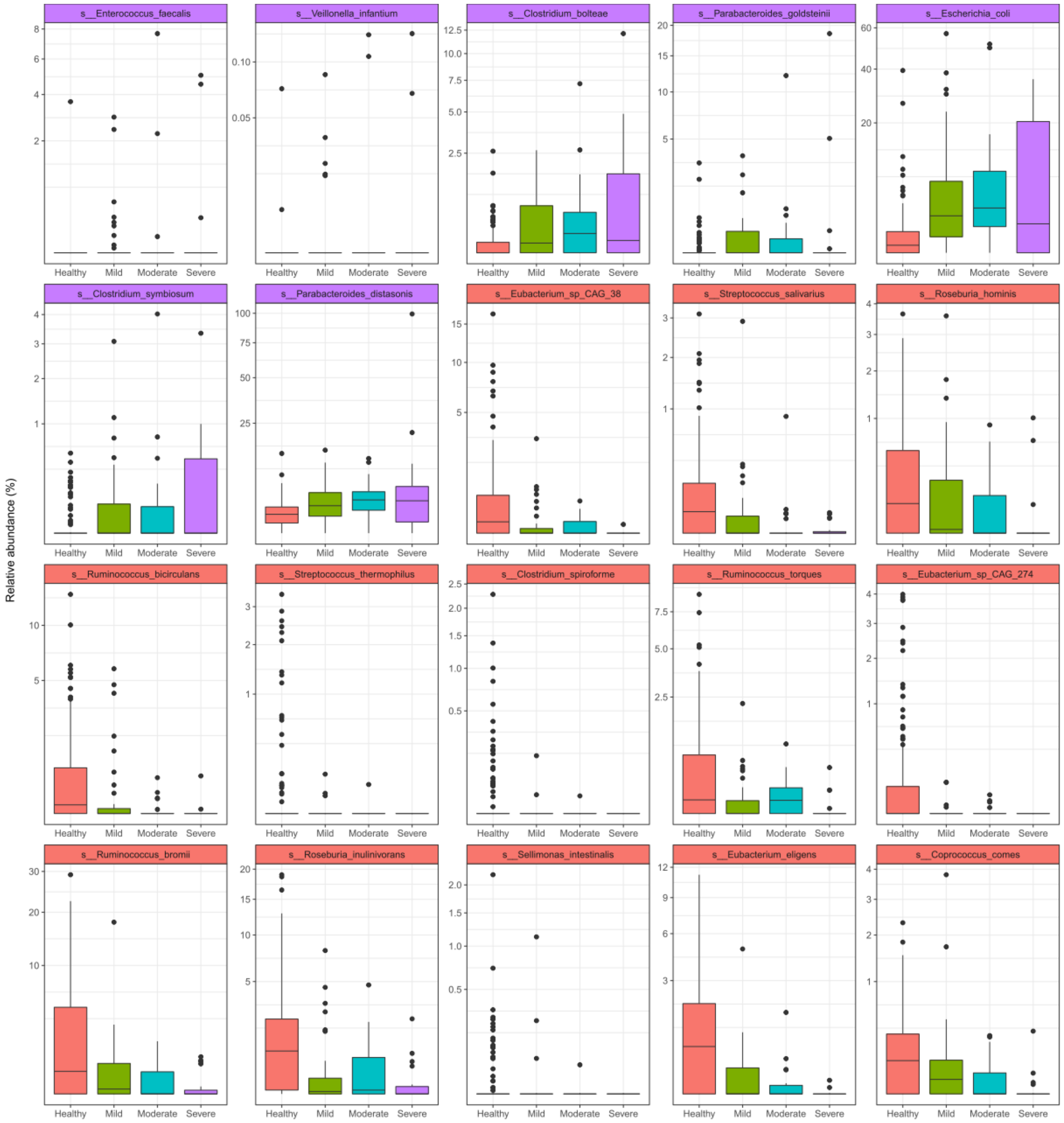

Supplementary Fig. 6| Gut species associate with AP severity. Boxplots showing the species that showed a continuous positive (n = 7) or negative (n = 13) trend in the severity. Boxes represent the interquartile range between the first and third quartiles and the median (internal line). Whiskers denote the lowest and highest values within 1.5 times the range of the first and third quartiles, respectively; dots represent outlier samples beyond the whiskers.

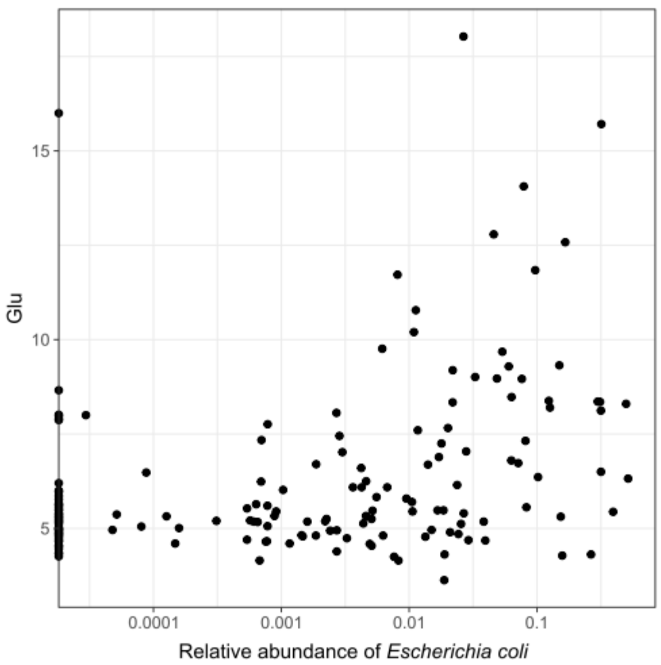

Supplementary Fig. 7| Scatter plot showing the correlation between *Escherichia coli* and fasting blood glucose level. Glu, fasting blood glucose level.

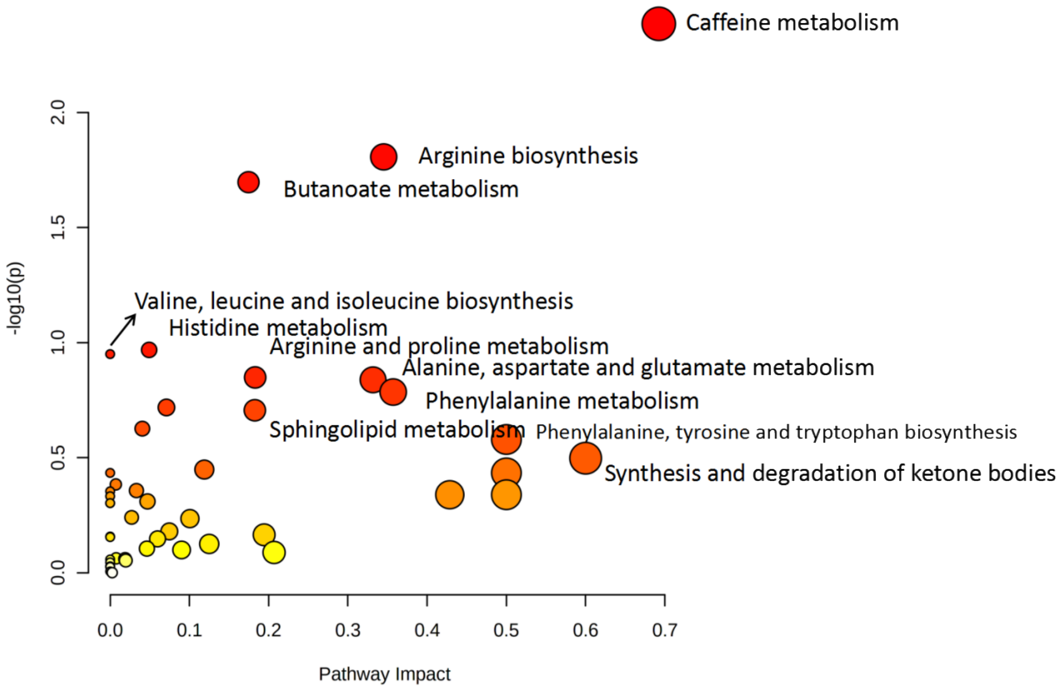

Supplementary Fig. 8| Metabolic pathway enrichment analysis. Metabolic pathway enrichment analysis based on the KEGG (Kyoto Encyclopedia of Genes and Genomes) database was performed to determine differentially enriched pathways between AP patients and healthy controls.

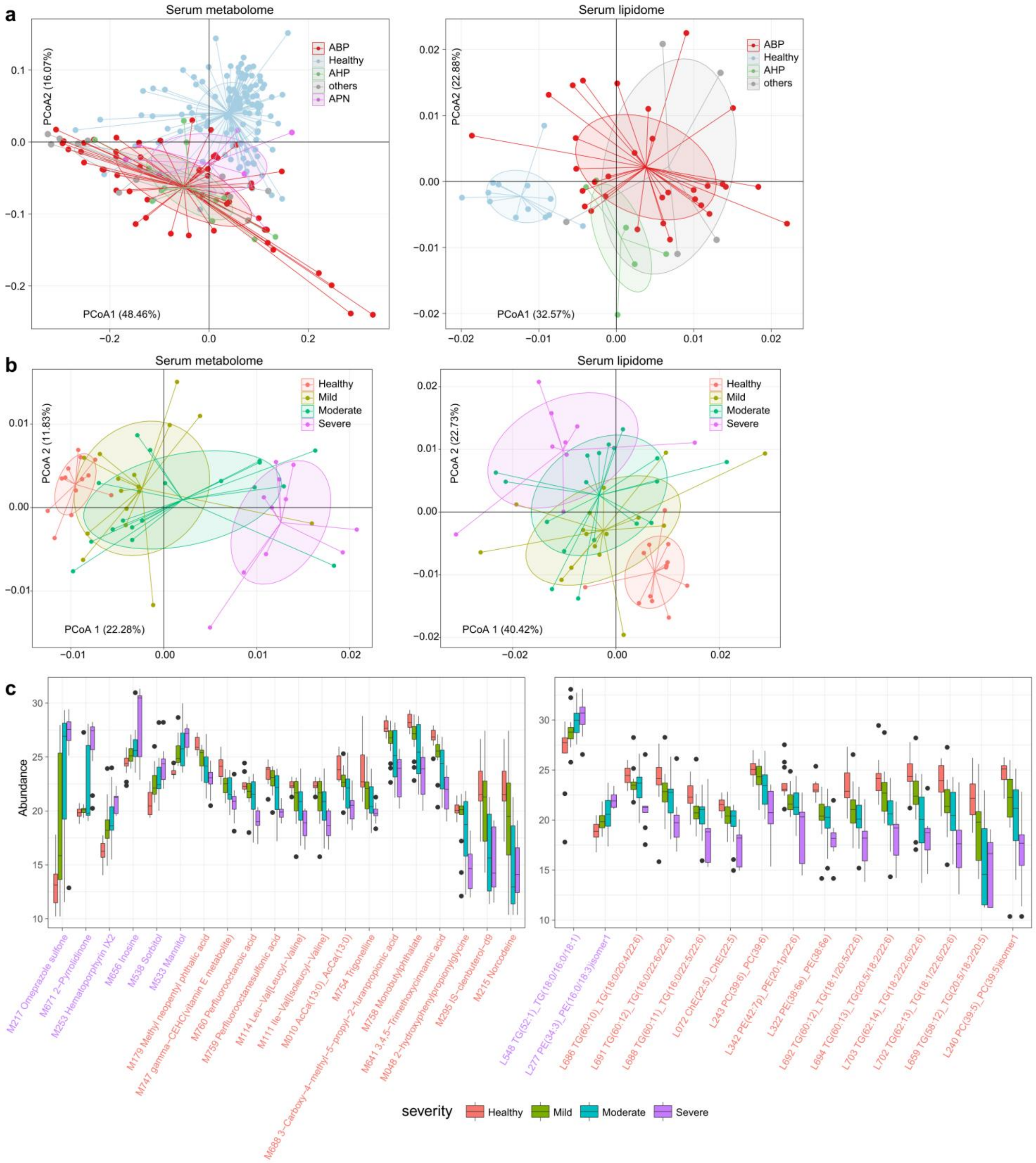

Supplementary Fig. 9| Differences in the serum metabolome and lipidome associated with AP etiology severity. (a-b) Principal coordinates analysis (PCoA) based on the Bray-Curtis dissimilarity of serum metabolome (left panel) and lipidome (right panel) between patients and controls, patients are grouped by AP etiology (a) and severity (b). The result is shown in the first two principal coordinates (PC1 and PC2), and the ratios of variance contributed by these two PCs are shown. Colored points represent the samples, and circles cover samples near the center of gravity for each group. QC, quality control samples. **(c)** Boxplots showing the serum metabolites (left panel) and lipids (right panel) that showed a continuous positive or negative trend in the severity. Boxes represent the interquartile range between the first and third quartiles and the median (internal line). Whiskers denote the lowest and highest values within 1.5 times the range of the first and third quartiles, respectively; dots represent outlier samples beyond the whiskers.

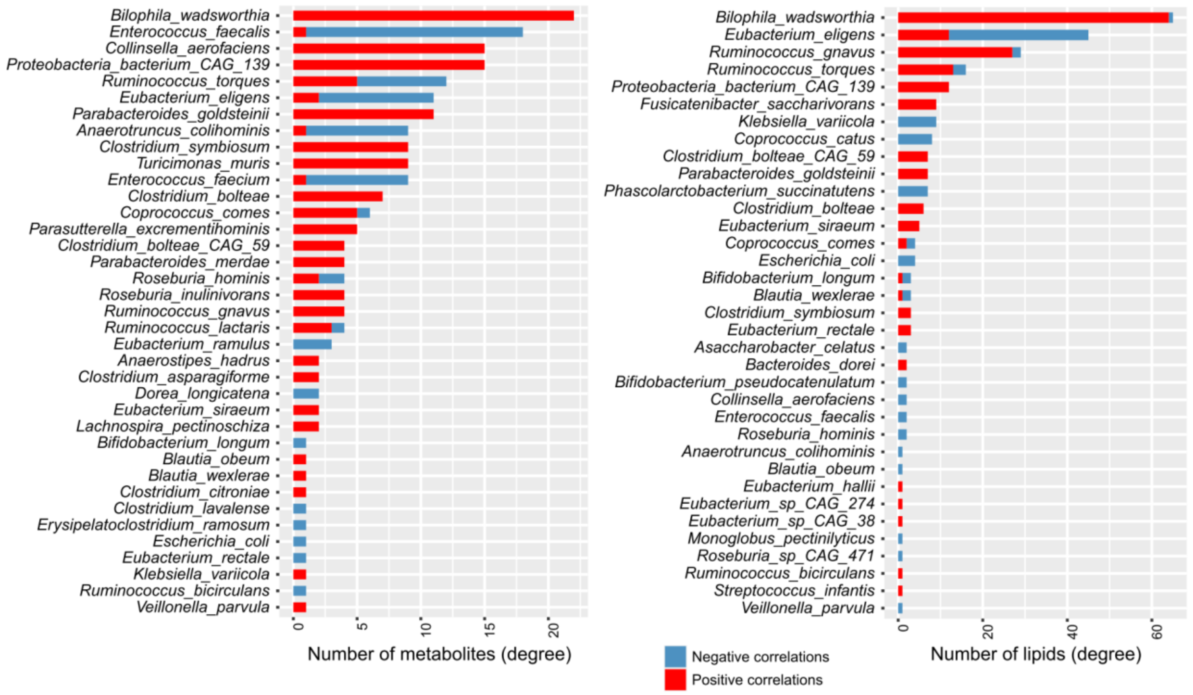

Supplementary Fig. 10| Correlations between gut species and serum metabolites and lipids. Barplots showing the number of correlations between AP-associated gut species and serum metabolites (left panel) and lipids (right panel). The Spearman correlation coefficient was used to evaluate the correlation, and correlations with an absolute correlation coefficient ρ>0.35 and a correlation test q<0.05 are shown in the network. Blue and red bars represent negative and positive correlations, respectively.

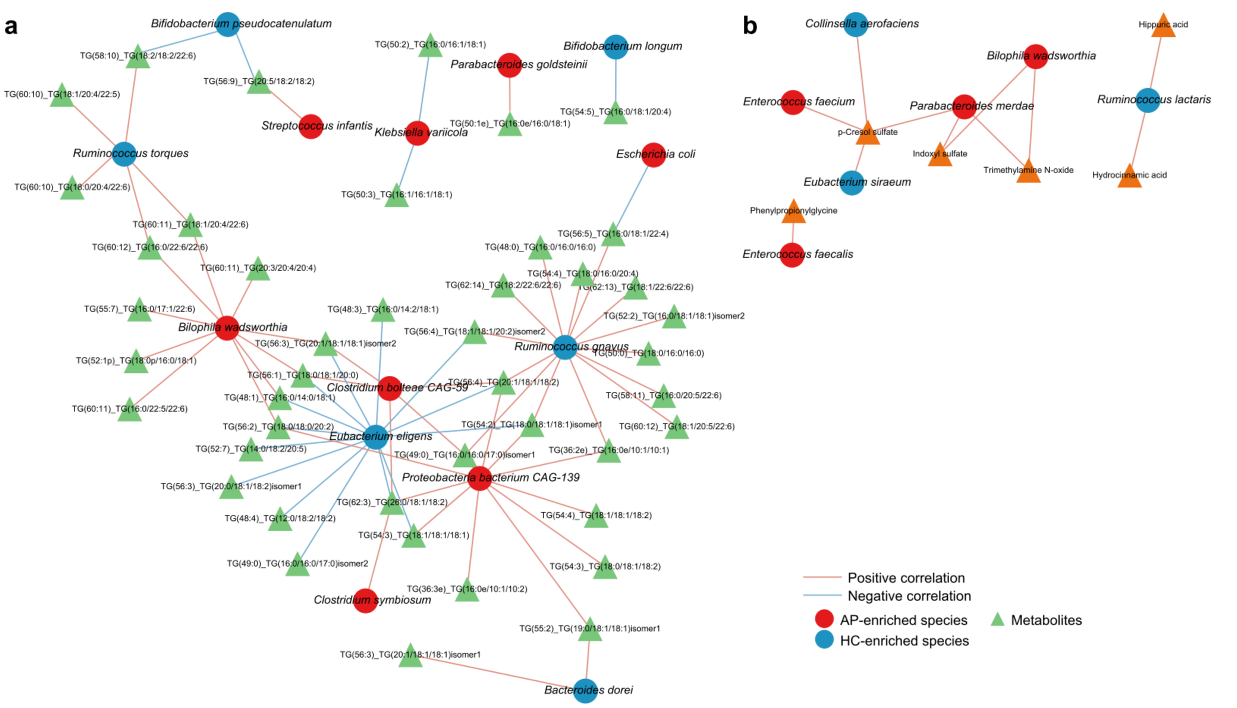

Supplementary Fig. 11| Correlations between gut species and serum TGs and microbial metabolites. Network showing the correlations between AP-associated gut species and serum TGs **(a)** and microbial metabolites **(b)**. The Spearman correlation coefficient was used to evaluate the correlation, and

correlations with an absolute correlation coefficient ρ>0.35 and a correlation test q<0.05 are shown in the network. Blue and red lines represent negative and positive correlations, respectively.

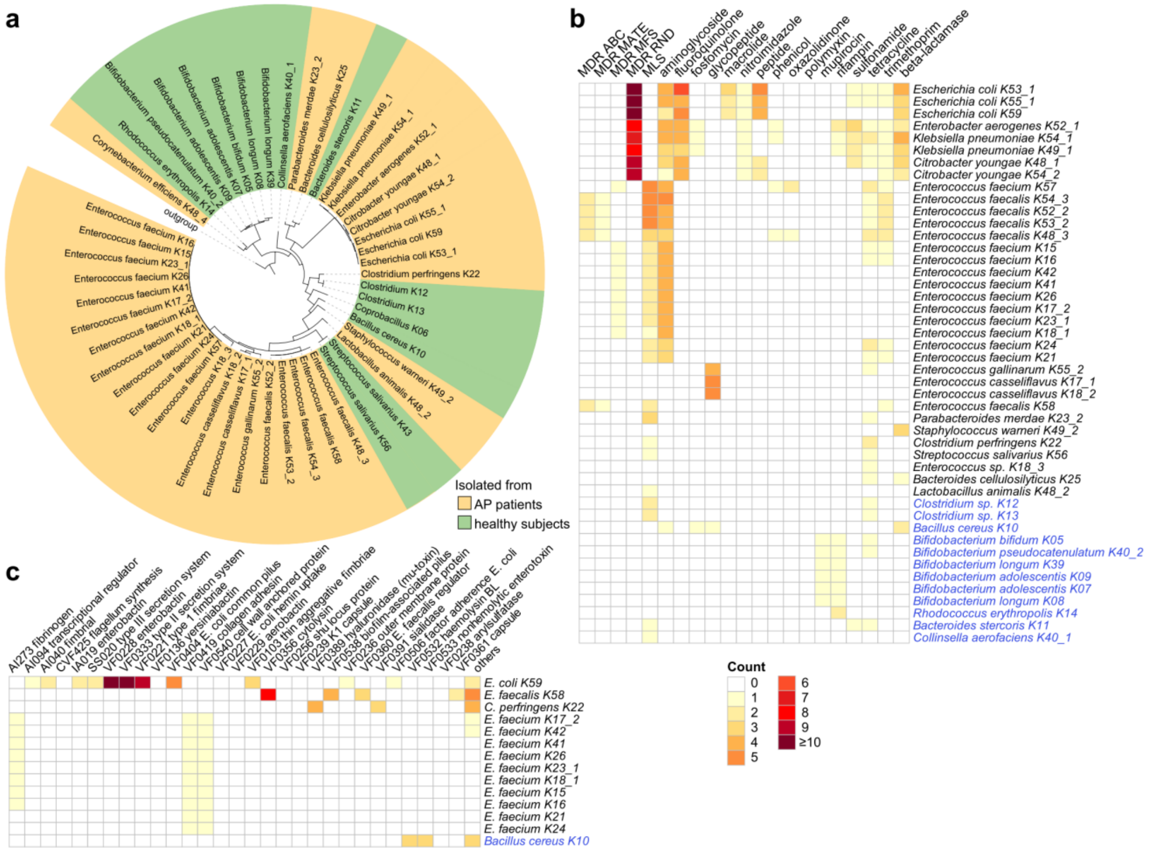

Supplementary Fig. 12| Distribution of antibiotic resistance genes and virulence-associated genes in gut isolates. (a) Phylogenetic representation of 49 bacterial strains isolated from AP patients and healthy controls. The maximum likelihood phylogenetic tree is inferred based on the marker genes of 49 strains, with *Akkermansia muciniphila* BAA-835 as outgroup species. **(b-c)** Heatmaps show the number of antibiotic resistance genes **(a)** and virulence-associated genes **(c)** for each strain. The strains isolated from healthy controls are labeled in blue.

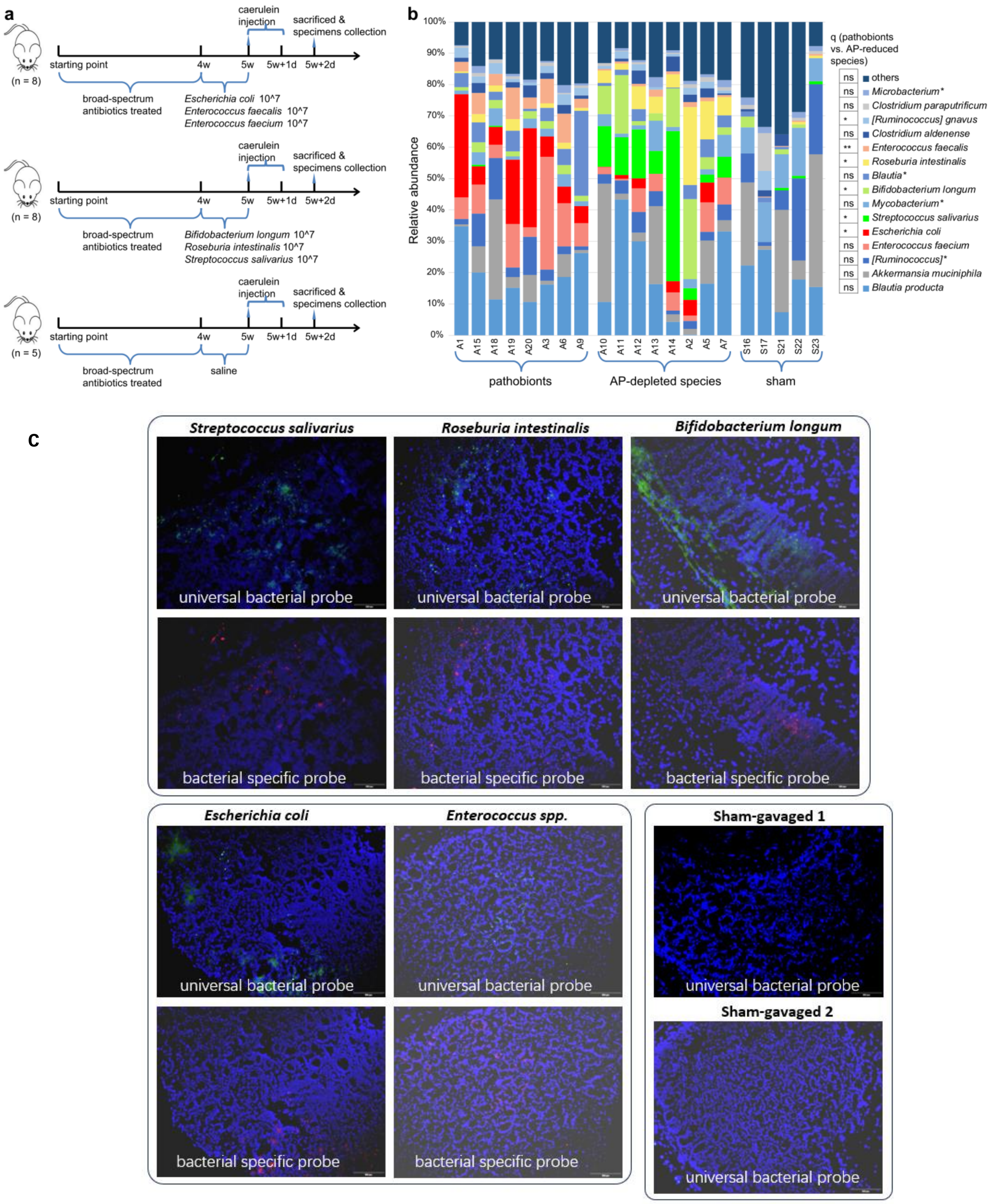

Supplementary Fig. 13| Animal experiment scheme. (a) Overview of the workflow for mouse experiment in this study. **(b)** Composition of the gut microbial communities in mice after 1-week gavage of pathobionts and AP-depleted species. Gut microbial abundances of mouse fecal samples are quantified by 16S rRNA amplicon sequencing analysis. Significance levels of the comparison of species between pathobionts- and AP-depleted species-gavaged mice are shown in the right boxes: *, $q<0.05$; **, $q<0.01$; ns, not significant; Wilcoxon rank-sum test. **(c)** Fluorescence in situ hybridization (FISH) detection of anaerobic bacteria in colon of pathobionts and AP-depleted species recipient mice. FISH are performed

using a FAM-conjugated universal bacterial probe (EUB338, green) and Cy3-conjugated specific probes for three bacteria (red). Scale bars, 100 μm. Two independent experiments were performed with consistent results.

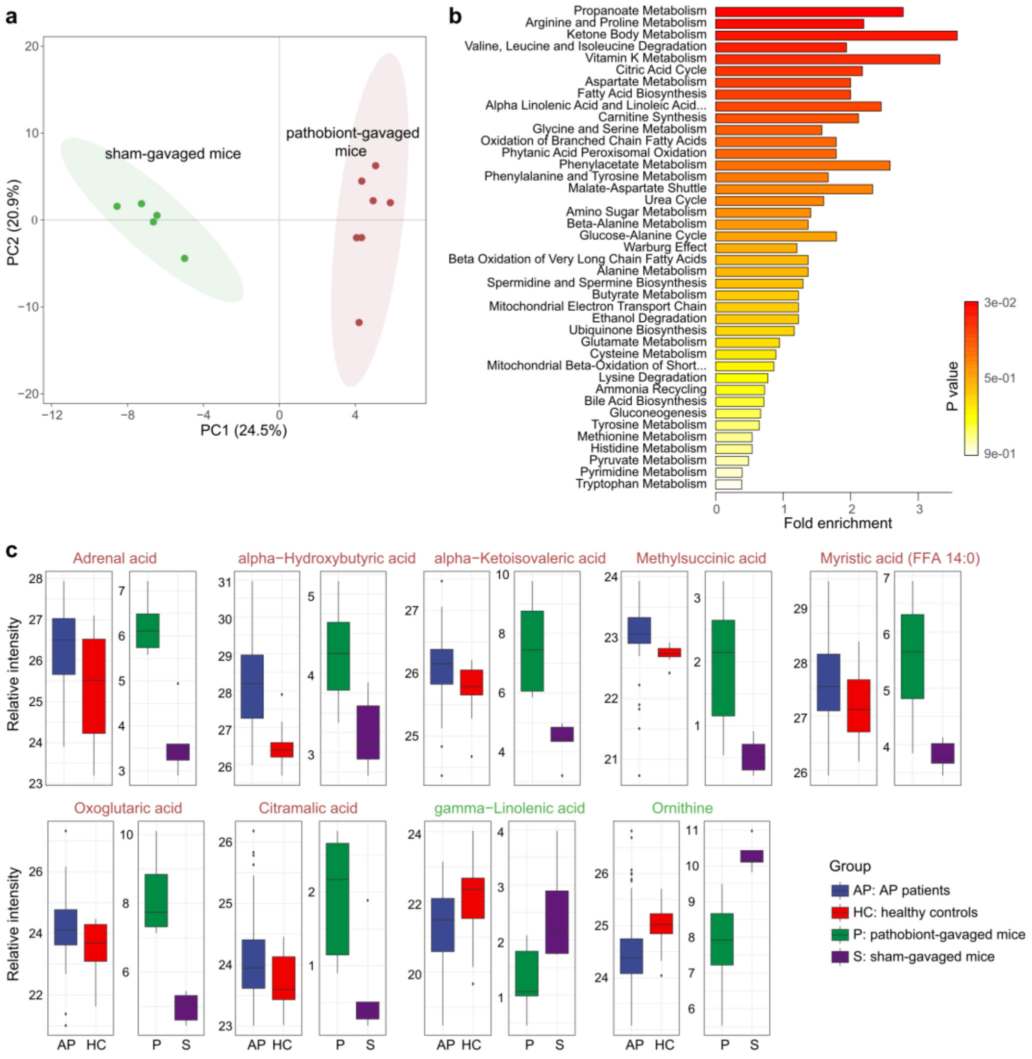

Supplementary Fig. 14| Comparison of the fecal metabolome between pathobiont-gavaged and sham-gavaged mice. (a) Orthogonal partial least squares discrimination analysis (OPLS-DA) score plots of metabolites between pathobiont-gavaged and sham-gavaged mice. The result is shown in the first two principal coordinates (PC1 and PC2), and the ratios of variance contributed by these two PCs are shown. **(b)** Metabolic pathway enrichment analysis based on the SMPDB (Small Molecule Pathway Database) database was performed to determine differentially enriched pathways between pathobiont-gavaged and sham-gavaged mice. **(c)** 9 metabolites displaying consistent trends were identified when comparing the alterations in the fecal metabolome of pathobiont-gavaged mice with the serum metabolic changes observed in AP patients. Boxes represent the interquartile range between the first and third quartiles and the median (internal line). Whiskers denote the lowest and highest values within 1.5 times the range of the first and third quartiles, respectively; dots represent outlier samples beyond the whiskers.
