# Supplementary material for: Integrative metagenomic and metabolomic analyses reveal the potential of gut microbiota to exacerbate acute pancreatitis
Source: NPJ Biofilms Microbiomes. 2024 Mar 21;10:29. doi: 10.1038/s41522-024-00499-4 (PMC10957925; doi:10.1038/s41522-024-00499-4)
Supplement: Supplementary file 3 — reporting-summary [file 41522_2024_499_MOESM3_ESM.pdf]

nature portfolio | reporting summary

nature portfolio

Corresponding author(s): Dong Shang

Last updated by author(s): Mar 9, 2024

Reporting Summary

Nature Portfolio wishes to improve the reproducibility of the work that we publish. This form provides structure for consistency and transparency in reporting. For further information on Nature Portfolio policies, see our Editorial Policies and the Editorial Policy Checklist.

Statistics

For all statistical analyses, confirm that the following items are present in the figure legend, table legend, main text, or Methods section.

n/a	Confirmed	
☐	☒	The exact sample size (*n*) for each experimental group/condition, given as a discrete number and unit of measurement
☐	☒	A statement on whether measurements were taken from distinct samples or whether the same sample was measured repeatedly
☐	☒	The statistical test(s) used AND whether they are one- or two-sided<br>*Only common tests should be described solely by name; describe more complex techniques in the Methods section.*
☐	☒	A description of all covariates tested
☐	☒	A description of any assumptions or corrections, such as tests of normality and adjustment for multiple comparisons
☐	☒	A full description of the statistical parameters including central tendency (e.g. means) or other basic estimates (e.g. regression coefficient) AND variation (e.g. standard deviation) or associated estimates of uncertainty (e.g. confidence intervals)
☐	☒	For null hypothesis testing, the test statistic (e.g. *F*, *t*, *r*) with confidence intervals, effect sizes, degrees of freedom and *P* value noted<br>*Give P values as exact values whenever suitable.*
☒	☐	For Bayesian analysis, information on the choice of priors and Markov chain Monte Carlo settings
☐	☒	For hierarchical and complex designs, identification of the appropriate level for tests and full reporting of outcomes
☐	☒	Estimates of effect sizes (e.g. Cohen's *d*, Pearson's *r*), indicating how they were calculated

Our web collection on statistics for biologists contains articles on many of the points above.

Software and code

Policy information about availability of computer code

Data collection	human reference sequences (GRCh38)<br>Comprehensive Antibiotic Resistance Database (CARD)<br>National Center for Biotechnology Information (NCBI) RefSeq database<br>NCBI Bacterial Antimicrobial Resistance Reference Gene Database<br>ABRicate (https://github.com/tseemann/abricate)<br>VFDB (Virulence Factor Database)
Data analysis	SPSS software<br>fastp<br>Bowtie 2<br>MetaPhlAn3<br>HUMAnN3<br>SPAdes<br>Quast<br>SpecI<br>Prodigal<br>PhyloPhlAn2<br>interactive Tree Of Life (iTOL)<br>ARG-ANNOT<br>ResFinder

R v3.3.2
R packages (vegan, fdrtool)

For manuscripts utilizing custom algorithms or software that are central to the research but not yet described in published literature, software must be made available to editors and reviewers. We strongly encourage code deposition in a community repository (e.g. GitHub). See the Nature Portfolio guidelines for submitting code & software for further information.

Data

Policy information about availability of data

All manuscripts must include a data availability statement. This statement should provide the following information, where applicable:

- Accession codes, unique identifiers, or web links for publicly available datasets
- A description of any restrictions on data availability
- For clinical datasets or third party data, please ensure that the statement adheres to our policy

The raw whole-metagenomic shotgun sequencing dataset acquired in this study has been deposited in the European Bioinformatics Institute (EBI) database under the accession code PRJEB36300. The assembled bacterial genome sequences reported in this article were deposited in the NCBI BioProject PRJNA612981. The metabolome datasets reported in this article were available at the MetaboLights database (https://www.ebi.ac.uk/metabolights/) with accession number MTBLS9696. Other data related to the current article are available from the corresponding author on reasonable request.

Research involving human participants, their data, or biological material

Policy information about studies with human participants or human data. See also policy information about sex, gender (identity/presentation), and sexual orientation and race, ethnicity and racism.

Reporting on sex and gender	This study contained 82 AP patients and 115 healthy controls matched gender, age and BMI.<br>Host properties such as sex, age, and body mass index showed minimal impact on the microbiota (effect size < 0.5%, PERMANOVA p > 0.05).
Reporting on race, ethnicity, or other socially relevant groupings	Not applicable
Population characteristics	The analysis focused on 197 fecal samples from 82 AP patients (comprising 38 mild AP [MAP], 19 moderately severe AP [MSAP], and 25 severe AP [SAP]) and 115 matched healthy controls. The demographic and clinical characteristics of the participants are summarized in Supplementary Table 1.
Recruitment	A total of 325 patients diagnosed with AP and 136 healthy subjects were recruited for this study. Following meticulous screening according to the inclusion and exclusion criteria and in conjunction with a random selection of a subset of mild and moderately severe patients based on a computer-generated randomization method (Supplementary Fig. 1),
Ethics oversight	This study received approval from the Ethics Committee of the First Affiliated Hospital of Dalian Medical University (YJ-KS-KY-2019-93), and all participants provided written informed consent to participate in the study.

Note that full information on the approval of the study protocol must also be provided in the manuscript.

Field-specific reporting

Please select the one below that is the best fit for your research. If you are not sure, read the appropriate sections before making your selection.

☒ Life sciences ☐ Behavioural & social sciences ☐ Ecological, evolutionary & environmental sciences

For a reference copy of the document with all sections, see nature.com/documents/nr-reporting-summary-flat.pdf

Life sciences study design

All studies must disclose on these points even when the disclosure is negative.

Sample size	A total of 325 patients diagnosed with AP and 136 healthy subjects were recruited for this study. Following meticulous screening according to the inclusion and exclusion criteria and in conjunction with a random selection of a subset of mild and moderately severe patients based on a computer-generated randomization method (Supplementary Fig. 1), the analysis focused on 197 fecal samples from 82 AP patients and 115 matched healthy controls.
Data exclusions	Patients were excluded if they had medical histories of gastrointestinal disorders, immune deficiency, and cancers. To minimize the impact of antibiotic or other drug treatment on the gut microbiota, patients who did not provide fecal samples within 48 hours after admission were excluded from the analysis. A computer-generated randomization sampling method, facilitated by the Rv.Uniform function in SPSS platform, was used to randomly select a subset of MAP and MSAP patients, as well as all SAP patients for further experiments (Supplementary Fig. 1).
Replication	Not applicable
Randomization	Not applicable

Blinding	Not applicable

Reporting for specific materials, systems and methods

We require information from authors about some types of materials, experimental systems and methods used in many studies. Here, indicate whether each material, system or method listed is relevant to your study. If you are not sure if a list item applies to your research, read the appropriate section before selecting a response.

Materials & experimental systems

n/a	Involved in the study
☒	☐ Antibodies
☒	☐ Eukaryotic cell lines
☒	☐ Palaeontology and archaeology
☐	☒ Animals and other organisms
☒	☐ Clinical data
☒	☐ Dual use research of concern
☒	☐ Plants

Methods

n/a	Involved in the study
☒	☐ ChIP-seq
☒	☐ Flow cytometry
☒	☐ MRI-based neuroimaging

Animals and other research organisms

Policy information about studies involving animals; ARRIVE guidelines recommended for reporting animal research, and Sex and Gender in Research

Laboratory animals	21 Six-week-old male C57BL/6 mice obtained from the Specific Pathogen Free Animal Center of Dalian Medical University were randomly divided into three groups
Wild animals	Not applicable
Reporting on sex	Six-week-old male C57BL/6 mice obtained from the Specific Pathogen Free Animal Center of Dalian Medical University were randomly divided into three groups
Field-collected samples	At 24 hours after the first injection of caerulein, the mice were anesthetized with inhaled isoflurane using a gas anesthesia machine, sera, pancreatic tissue, and different intestinal segments (ileum, cecum, and colon) were obtained and sacrificed animals after sampling.
Ethics oversight	All animal experiments were performed in accordance with the recommendations of the Guide for the Care and Use of Laboratory Animals of the National Institute of Health. The animal protocols were approved by the Committee on the Ethics of Animal Experiments of Dalian Medical University (No. AEE19001).

Note that full information on the approval of the study protocol must also be provided in the manuscript.

Plants

Seed stocks	Not applicable
Novel plant genotypes	Not applicable
Authentication	Not applicable
